# Metal–organic framework (MOF) hybridized gold nanoparticles as a bifunctional nanozyme for glucose sensing†

**DOI:** 10.1039/d3sc02598e

**Published:** 2023-06-22

**Authors:** Pei-Hong Tong, Jing-Jing Wang, Xi-Le Hu, Tony D. James, Xiao-Peng He

**Affiliations:** a Key Laboratory for Advanced Materials and Joint International Research Laboratory of Precision Chemistry and Molecular Engineering, Feringa Nobel Prize Scientist Joint Research Center, School of Chemistry and Molecular Engineering, East China University of Science and Technology 130 Meilong Rd. Shanghai 200237 China xphe@ecust.edu.cn; b The International Cooperation Laboratory on Signal Transduction, Eastern Hepatobiliary Surgery Hospital, National Center for Liver Cancer Shanghai 200438 China; c Department of Chemistry, University of Bath Bath BA2 7AY UK t.d.james@bath.ac.uk; d School of Chemistry and Chemical Engineering, Henan Normal University Xinxiang 453007 China

## Abstract

Inspired by natural enzymes that possess multiple catalytic activities, here we develop a bifunctional metal–organic frame-work (MOF) for biosensing applications. Ultrasmall gold nano-particles (AuNPs) are grown in the internal cavities of an iron (Fe) porphyrin-based MOF to produce a hybridized nanozyme, AuNPs@PCN-224(Fe), in which AuNPs and PCN-224(Fe) exhibit the catalytic activity of glucose oxidase (GOx) and horseradish peroxidase (HRP), respectively. We established that the bifunctional nanozyme was capable of a cascade reaction to generate hydrogen peroxide in the presence of d-glucose and oxygen *in situ*, and subsequently activate a colorimetric or chemiluminescent substrate through HRP-mimicking catalytic activity. The nanozyme was selective over a range of other saccharides, and 93% of the catalytic activity was retained after being recycled five times.

## Abstract 2

Inspired by natural enzymes that possess multiple catalytic activities, here we develop a bifunctional metal–organic frame-work (MOF) for biosensing applications.

## Graphical abstract

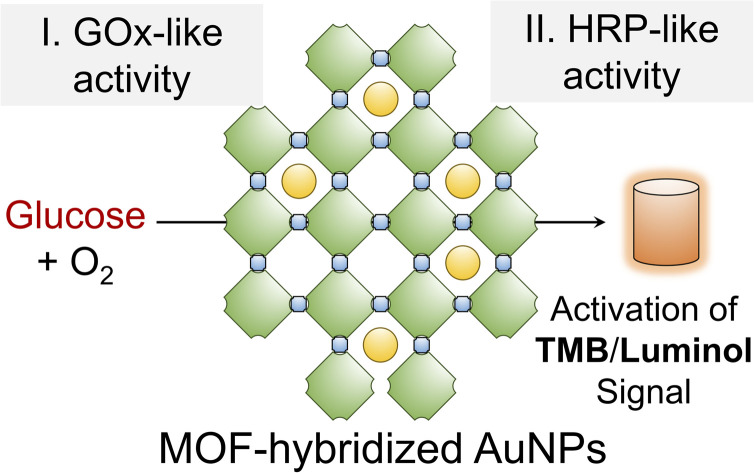

## Introduction

Nanozymes are artificial enzymes that exhibit enzyme-like activities.^1,2^ They are attractive alternatives to natural enzymes with superior properties in terms of catalytic activity, stability, recyclability, and cost,^3,4^ and have wide-range applications in the field of industrial catalysis, biosensing and biomedicine.^5–8^ Among the various nanozymes reported, peroxidase-like nanozymes based on metal and metal oxide-based nanomaterials,^9,10^ carbon-based nanomaterials,^11^ metal sulfides-based nanomaterials,^12^ and metal organic frameworks (MOFs)^13^ have been the most widely developed. MOFs are advanced materials constructed by the coordination chemistry between metal ions/clusters and organic ligands with size-tunable pores for substrate diffusion. The metal–ligand complexes inside MOFs can serve as catalytic sites to accelerate chemical reactions.^14,15^ For example, iron (Fe)-containing MOFs including MIL-100,^16^ MIL-101,^17^ MIL-53 ^18^ and MIL-88NH_2_,^19^ and metalloporphyrin-based MOFs such as PCN-224,^20^ PCN-600 ^21^ and PCN-222 ^22^ have been shown to exhibit peroxidase-like activities.

Natural enzymes that incorporate multiple catalytic activities are able to facilitate cascade reactions. For example, fatty acid biosynthesis is achieved by a single homodimeric fatty acid synthase bearing multiple catalytically active sites.^23,24^ In addition, acetyl coenzyme A (CoA) carboxylases catalyze the carboxylation of acetyl-CoA to produce malonyl-CoA, a key substrate involved in fatty acid biosynthesis, through the sequential action of the two active sites.^25,26^ Enlightened by this, hybrid materials created through the incorporation of MOFs with other functional materials have been developed, achieving the effective production of a number of value-added compounds *via* sequential catalytic processes.^27–32^ Among the vast variety of functional materials, gold nanoparticles (AuNPs) have been extensively used for biomedical applications.^33–36^ They also exhibit glucose oxidase (GOx)-like activity converting glucose molecules to hydrogen peroxide (H_2_O_2_).^37–40^ This has spurred the construction of AuNPs-based hybrid materials as nanozymes for disease diagnosis and therapy.^41–45^ However, MOF-based nanozymes incorporating AuNPs have been rarely developed.^46,47^

**Fig. 1:**
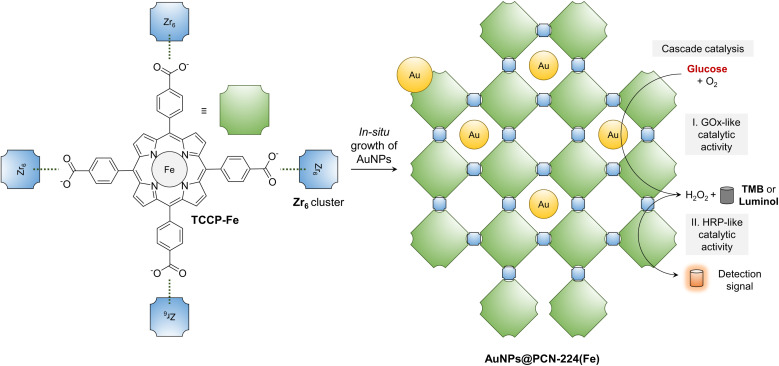
Schematic illustration of the construction of a bifunctional nanozyme, AuNPs@PCN-224(Fe), through the *in situ* growth of AuNPs inside a MOF formed by the coordination between Fe(ii) *meso*-tetra(4-carboxyphenyl)porphine (TCCP-Fe) and zirconyl chloride octahydrate (Zr_6_ cluster) for the cascade catalysis of d-glucose to produce colorimetric and chemiluminescent signals. TMB = 3,3′,5,5′-tetramethylbenzidine.

Here, given that the peroxidase activity of Fe-containing MOFs catalytically activates colorimetric and chemiluminescent substrates commonly used for serological diagnosis in the presence of H_2_O_2_, we sought to develop a new bifunctional nanozyme based on the hybridization between MOFs and AuNPs (Fig. 1). PCN-224(Fe) (Porous Coordination Network, which is synthesized by coordination between Zr_6_ clusters and Fe(ii) *meso*-tetra(4-carboxyphenyl)porphine) that mimics the catalytic activity of horseradish peroxidase (HRP) was constructed, where ultrasmall AuNPs were grown *in situ*. The resulting bifunctional AuNPs@PCN-224(Fe) exhibited a cascade reaction that involves the *in situ* generation of H_2_O_2_ in the presence of d-glucose and oxygen, and then activates 3,3′,5,5′-tetramethylbenzidine (TMB, a commercial colorimetric indicator) and Luminol (a commercial chemiluminescent indicator) substrates by an HRP-mimicking catalytic process.

## Results and discussion

### Synthesis and characterization of the nanozyme

To begin with, the Fe-containing MOF with peroxidase-like catalytic activity was synthesized. TCPP-Fe, an analog of the mononuclear Fe(ii) active site in natural ferroheme, was synthesized as the ligand (Scheme S1†). Then, TCPP-Fe was mixed with a Zr_6_ cluster in *N*,*N*-dimethylformamide and heated at 90 °C for 5 h, producing the MOF (PCN-224(Fe)) through coordination between the carboxylic acid groups in TCPP-Fe and the metal cluster.^48^ To construct the bifunctional nanozyme, HAuCl_4_ was used as the Au source for the *in situ* growth of AuNPs inside the pores of PCN-224(Fe).^6,49,50^

Fourier-transform infrared (FT-IR) spectroscopy was used to analyze the MOFs synthesized (Fig. S1†). We found that the asymmetric vibrational absorption intensities of C–OH and C=O of PCN-224(Fe) was much lower than those of TCPP, which suggests the coordination between Zr_6_ cluster and the carboxyl groups of the ligands. A symmetric Fe–N stretching at around 1000 cm^−1^ emerged, indicating the coordination of TCPP with Fe^2+^. While, the N–H bond absorption of PCN-224(Fe) diminished.

**Fig. 2:**
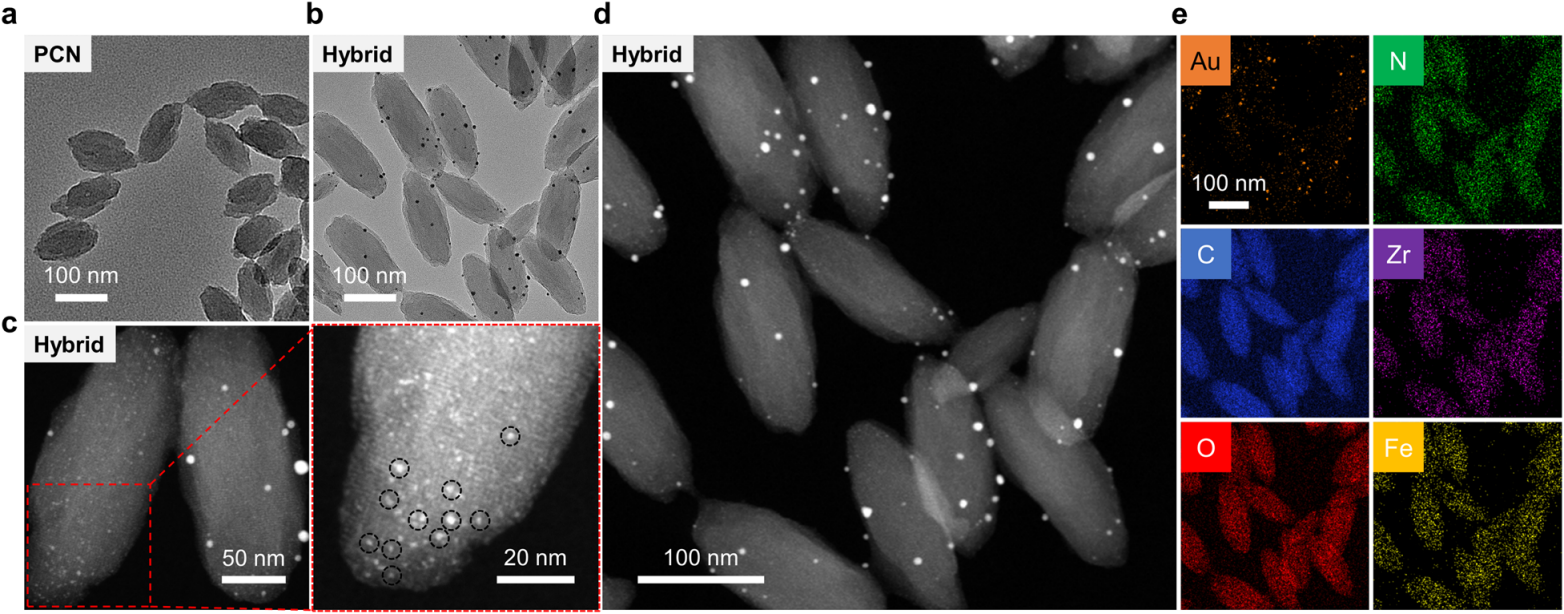
(a) TEM image of PCN-224(Fe). (b) TEM image of the AuNPs@PCN-224(Fe) (Hybrid). (c and d) Dark-field TEM images of Hybrid at different scales; the red framed image is an enlarged area of the image. (e) Elemental mapping images of the Hybrid.

Scanning electron microscopy (SEM) and transmission electron microscopy (TEM) were used to characterize the morphology of the MOFs constructed. In the typical SEM (Fig. S2a†) and TEM (Fig. 2a) images of PCN-224(Fe), uniform lenticular species were detected with an average size of 100 nm. This morphological feature agrees with previously reported PCN-224(Fe) MOFs.^51^ In contrast, the morphology of PCN-224 appeared to be more spherical than that of PCN-224(Fe) (Fig. S2b†). This morphological difference between PCN-224(Fe) and PCN-224 could be ascribed to the coordination of TCPP with Fe that substantially enhances the rigidity of the self-assembled MOF construct. Moreover, after coordination with Fe ions, the size of PCN-224 slightly decreased, suggesting that Fe ions could also inhibit the growth of the MOFs.^48,51–53^ Energy-dispersive X-ray (EDX) (Fig. S3†) mapping confirmed that Zr, Fe, C, O, and N elements are uniformly distributed in PCN-224(Fe). Similarly, a lenticular morphology was seen for AuNPs@PCN-224(Fe) with an average size of 150 nm (Fig. 2b).

In the representative dark-field TEM (Fig. 2c and S4†) images of AuNPs@PCN-224(Fe), an average size of ∼1.5 nm was determined for the AuNPs embedded in the cavities of PCN-224(Fe) (Fig. S5a†). The uniform, ultrasmall sizes of these AuNPs are the result of a confinement effect by the cavities of PCN-224(Fe). However, relatively large AuNPs could be seen on the surface of PCN-224(Fe). These larger AuNPs were generated because of a lack of constrained growth outside the cavities of PCN-224(Fe). Considering both the internal and surface-attached AuNPs, the general size distribution of AuNPs was adjusted by the Gaussian function with an average particle diameter of ∼2.7 nm being determined (Fig. S5b†). Subsequently, EDX mapping indicated that these particles are AuNPs (Fig. 2d and e), suggesting the successful construction of the AuNPs-hybridized MOF nanozyme. Quantitative analysis of the EDX result shown in Fig. S6† suggests a mass fraction of AuNPs in the hybrid of ∼2%.

To better characterize AuNPs@PCN-224(Fe), powder X-ray diffraction (PXRD), nitrogen adsorption analysis (NAA), and X-ray photoelectron spectroscopy (XPS) were used. The PXRD pattern of the as-prepared PCN-224(Fe) and AuNPs@PCN-224(Fe) powder matched the simulated pattern of PCN-224(Fe) (Fig. S7†). The main diffraction peaks observed at 6.494° and 7.956° are the [022] and [222] crystal plane, respectively.^51^ The PXRD data shows that the crystalline state of AuNPs@PCN-224(Fe) is identical to that of PCN-224(Fe), suggesting that the presence of the *in situ* grown AuNPs did not alter the configuration of the MOF. The NAA analysis determined the Brunauer–Emmett–Teller (BET) surface area of AuNPs@PCN-224(Fe) to be 76.5256 m^2^ g^−1^, which is ∼7-fold smaller than that of PCN-224(Fe) (525.3523 m^2^ g^−1^), suggesting that the AuNPs existed in the cavities of the former inhibited nitrogen adsorption (Fig. S8†). The elemental components and valence states of PCN-224(Fe) and AuNPs@PCN-224(Fe) were determined by XPS. The survey spectra indicated that both materials contain Zr, C, N, O and Fe, and the hybrid contains an additional Au element (Fig. S9a and f†). Two Fe species in PCN-224(Fe) at 711.2 and 724.1 eV (Fig. S9b†) were seen in the XPS spectrum of Fe 2p, which are assigned to Fe 2p_3/2_ and Fe 2p_1/2_, respectively.^54^ The binding energies emerged at 398.7 eV and 401.1 eV in the N 1s spectrum are assigned to the pyrrolic =N– and N–H, respectively (Fig. S9c†).^55^ In addition, binding energies at 182.9 eV and 185.3 eV, assigned to Zr 3d_3/2_ and Zr 3d_1/2_, respectively (Fig. S9d†), and that at 531.8 eV assigned to O 1s of the hydroxyl groups, were detected (Fig. S9e†).^56^ The Fe 2p spectrum of AuNPs@PCN-224(Fe) (Fig. S9g†) is consistent with that of PCN-224(Fe), and in the Au spectrum of the hybrid, Au 4f_7/2_ and Au 4f_5/2_ peaks at 84.4 and 88.1 eV were observed, respectively (Fig. S9h†). This suggests the presence of metallic Au^0^ species belonging to AuNPs in the nanozyme.^46^

Spectroscopic techniques were also used to characterize AuNPs@PCN-224(Fe). In the UV-vis spectrum of TCPP, the Soret band at ∼420 nm and the four Q bands at 515 nm, 550 nm, 590 nm and 650 nm, characteristic of porphyrins, were visible (Fig. S10a†). After coordination with Fe^2+^, the Q bands of TCPP-Fe decreased due to the increased symmetry of molecular structure. The Soret band of PCN-224(Fe) (418 nm) slightly redshifted with respect to TCPP-Fe (405 nm). The ∼13 nm redshift could be attributed to the hydrophobic property of the octahedral cavity formed in the MOFs and the sensitivity of the Soret band to the dielectric constant of the solvent.^57,58^ As shown in Fig. S10b,† a localized surface plasmon resonance (LSPR) band of the AuNPs was seen at ∼527 nm. In contrast, no LSPR signal was detected for AuNPs@PCN-224(Fe), which suggests an ultra-small size of the AuNPs grown in the MOFs.^59,60^ In its surface-enhanced Raman scattering (SERS) spectrum, Raman shifts at 1496/1550 cm^−1^ and 1232/1355 cm^−1^, characteristic of the C–C stretching and the deformation of the pyrrole ring of PCN-224(Fe) were detected, respectively (Fig. S11†).^47,48^ While the shape of the Raman spectrum for AuNPs@PCN-224(Fe) did not change with respect to that of PCN-224(Fe), the intensity was largely enhanced. This was due to the interparticle plasmon coupling effect of AuNPs, giving rise to an enhancement of the electromagnetic field, and thus an intensified SERS signal.^49^

### Measurement of the catalytic activities of the nanozyme

**Fig. 3:**
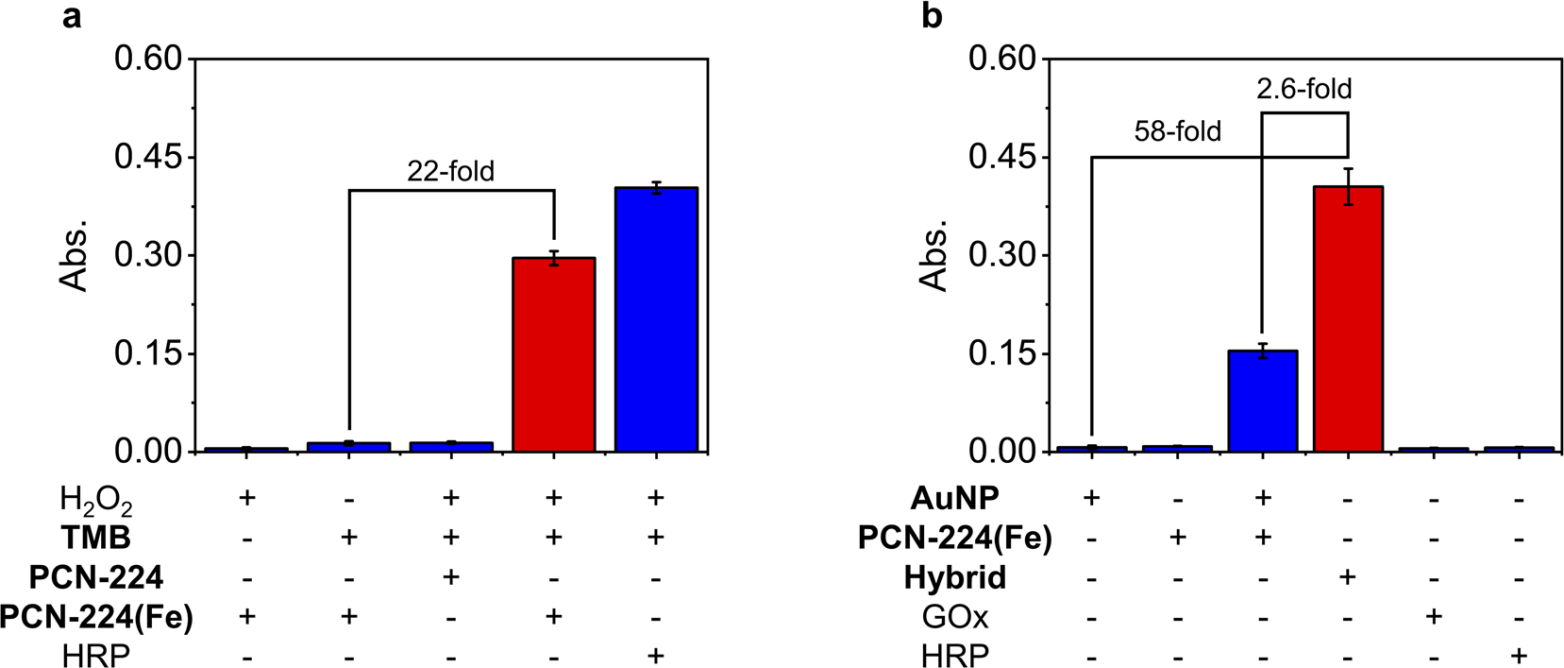
(a) Analysis of the peroxidase activity of PCN-224(Fe) by measuring the UV-vis absorbance (at 652 nm) of TMB in PBS solution (37 °C, 10 min) under the various indicated conditions. The concentrations used for H_2_O_2_, TMB, PCN-224, PCN-224(Fe) and HRP are 5 mM, 5 mM, 100 μg mL^−1^, 100 μg mL^−1^ and 100 μg mL^−1^, respectively. (b) Analysis of the dual enzymatic activities of AuNPs@PCN-224(Fe) (Hybrid) by measuring the UV-vis absorbance (at 652 nm) of TMB in PBS (37 °C, 40 min) under the various indicated conditions. The concentrations used for AuNP, PCN-224(Fe), Hybrid, GOx and HRP are 20 μg mL^−1^, 0.98 mg mL^−1^, 1 mg mL^−1^, 20 μg mL^−1^ and 0.98 mg mL^−1^, respectively. PBS = phosphate buffered saline.

The peroxidase-like activity of PCN-224(Fe) was first evaluated using TMB as a colorimetric substrate in the presence of H_2_O_2_. The oxidized form of TMB exhibiting a characteristic UV-vis absorption peak at 652 nm was monitored by a spectrometer. Shown in Fig. 3a are the absorption changes of TMB at 652 nm under various conditions. We found a significant absorption enhancement (∼22-fold) of TMB in the presence of PCN-224(Fe) and H_2_O_2_. A similar phenomenon was also observed when HRP was used instead of PCN-224(Fe) under the same conditions, suggesting that the MOF material mimics the natural enzyme to catalytically oxidize TMB. However, the absorption enhancement was minimal when the metal ion is absent (as in the case of PCN-224), indicating the importance of Fe in the MOF system for the catalysis. In addition, when either H_2_O_2_ or TMB was absent, the catalytic action failed to take place. We also determined that the absorption enhancement of TMB is dependent on both the concentration of PCN-224(Fe) (Fig. S12a†) and H_2_O_2_ (Fig. S12b†) added.

Next, the cascade catalytic activity of the nanozyme, AuNPs@PCN-224(Fe), was determined using TMB as the substrate and d-glucose as the analyte. To our delight, the mixture of AuNPs@PCN-224(Fe) with d-glucose activated the colorimetric signal of TMB by 58-fold (Fig. 3b). In contrast, when PCN-224(Fe) or AuNP (gold nanoparticles synthesized by the chemical reduction-method using sodium borohydride) were used instead of the nanozyme, the absorption of TMB remained almost unchanged. Likewise, when just GOx or HRP were used, the presence of d-glucose did not cause the color of TMB to change. Interestingly, when PCN-224(Fe) and AuNP are simply mixed, a slight enhancement in the absorption of TMB was detected. However, this enhancement was 2.6-fold smaller than that with AuNPs@PCN-224(Fe) as the catalyst. Moreover, the absorption enhancement of TMB with the nanozyme was also found to depend on the concentration of d-glucose (Fig. S13†). These results suggest that the hybridized nanozyme mimics the catalytic activity of both GOx and HRP to facilitate a cascade reaction, resulting in the oxidation of TMB.

A commercial chemiluminescent substrate, Luminol, was also used to test the cascade catalytic activity of AuNPs@PCN-224(Fe). Likewise, we found that the presence of just PCN-224(Fe) or AuNPs@PCN-224(Fe) did not elicit the chemiluminescence (CL) of the substrate (Fig. S14†). However, when H_2_O_2_ and d-glucose was added to the Luminol/PCN-224(Fe) and Luminol/AuNPs@PCN-224(Fe) system, a significant CL intensity increase of 23-fold and 18-fold was detected, respectively. This suggests that our MOF-based nanozyme is also suitable for CL-based detection assays.

To investigate the mechanism by which the substrates are activated by the nanozyme, electron-spin resonance (ESR) spectroscopy was employed. According to a previous report, the ·OH radical is the main reactive species to activate TMB and Luminol-based substrates.^61,62^ Therefore, we used 5,5-dimethyl-1-pyrroline-*N*-oxide (DMPO) as a trapping agent for ·OH. We observed quartet signals with relative intensities of 1 : 2 : 2 : 1, characteristic of DMPO-OH species, in a solution of PCN-224(Fe)/H_2_O_2_/DMPO (Fig. S15a†) and AuNPs@PCN-224(Fe)/d-glucose/DMPO (Fig. S15b†). This corroborates the production of ·OH radicals in the catalytic systems.

According to the results obtained from EPR analyses and previous literature reports,^46,63,64^ plausible mechanisms for substrate activation were proposed (Scheme S2†). When glucose molecules are transported into the inner cavities of AuNPs@PCN-224(Fe) through the mesoporous channel of PCN-224(Fe), they are catalyzed by the embedded AuNPs to generate H_2_O_2_ species. Then, the H_2_O_2_ is catalyzed by the surrounding PCN-224(Fe) backbone to produce ·OH radicals as evidenced by ESR spectroscopy. The resulting ·OH radicals react with Luminol to form electronically excited 3-aminophthalate anions (3-APA*), which then relaxes to the ground state by emitting light.^65^ In a similar manner, TMB is oxidized by ·OH radicals to produce a charge–transfer complex with a blue color being produced.^66^

Next, we measured the stability of the nanozyme. First, we found that the hydrodynamic diameter of AuNPs@PCN-224(Fe) gradually increased over a storage period of seven days (from ∼200 nm to ∼300 nm at the 7th day) (Fig. S16a†). This is common for MOF-based materials since they undergo slight aggregation when dispersed in aqueous solutions.^67,68^ Measuring the catalytic activity over the seven-day period indicated that ∼90% and ∼85% activity was retained when the nanozyme was stored at 4 °C and 25 °C for 7 days, respectively (Fig. S16b†). Subsequently, tests indicated that the nanozyme worked well over a wide pH range of 4.0–9.0 (Fig. S16c†) and a temperature range of 20 °C to 70 °C (Fig. S16d†). These attributes are favorable for practical use since the catalytic activity of most natural enzymes are very dependent on pH and temperature. The recyclability of AuNPs@PCN-224(Fe) was also determined. The result indicated that cascade catalytic activity of the nanozyme remained at 93% even after five cycles of recycling (Fig. S17†), establishing a unique feature that is absent for natural enzymes.

### Kinetic evaluation of the nanozyme

**Fig. 4:**
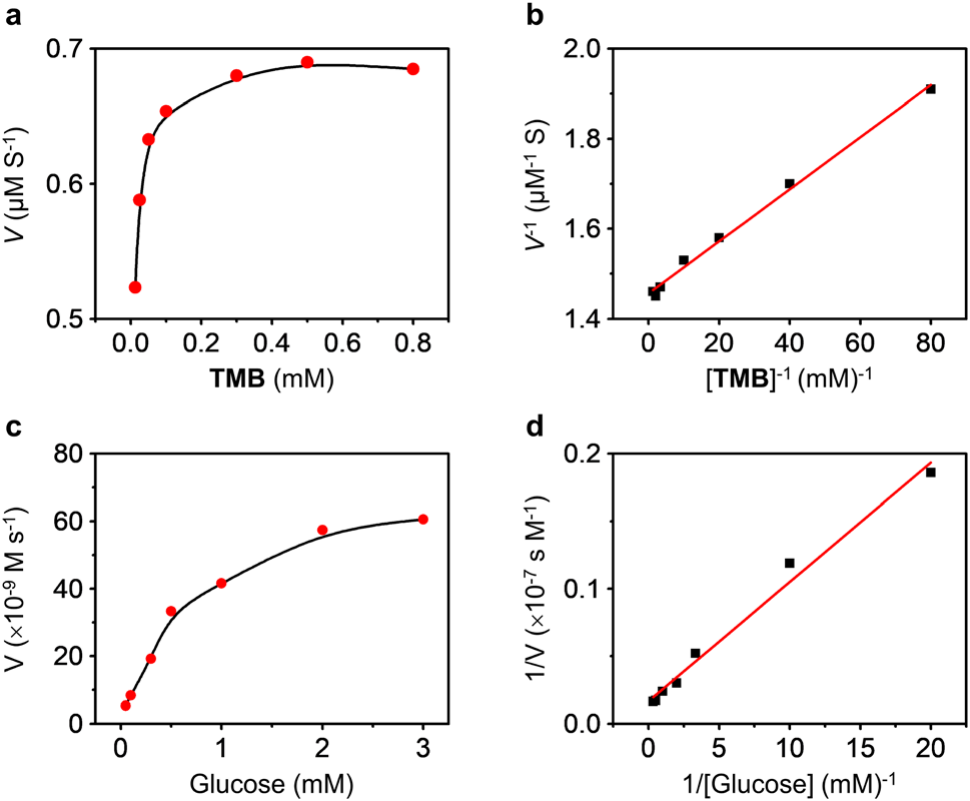
Steady-state kinetic analysis of the peroxidase activity of PCN-224(Fe) by (a) plotting the initial rate (*V*) of the catalytic reaction as a function of TMB concentrations, and (b) Lineweaver–Burk plots of the double reciprocal of the Michaelis–Menten equation. Steady-state kinetic analysis of cascade catalytic activity of AuNPs@PCN-224(Fe) by (c) plotting the initial rate of the catalytic reaction as a function of d-glucose concentrations, and (d) Lineweaver–Burk plots of the double reciprocal of the Michaelis–Menten equation.

Next, we evaluated the kinetics of the MOF-based nanozyme. We first measured continuously the absorption changes of TMB in the presence of PCN-224(Fe), and the Michaelis–Menten curve of the kinetic process was produced in Fig. 4a. Then, the Michaelis constant (*K*_m_) and maximum initial velocity (*V*_max_) of PCN-224(Fe) were obtained using the Lineweaver–Burk method (Fig. 4b). To better illustrate the catalytic activity of PCN-224(Fe), *K*_w_, which is a measure of the rate at which a catalyst catalyzes a reaction, was calculated according to the following equation: *K*_w_ = *V*_max_/*w*, where *V*_max_ and *w* are the maximal reaction velocity and mass concentration of the catalyst, respectively.^69^ A *V*_max_ of 0.687 μM s^−1^, *K*_m_ of 0.039 mM, and *K*_w_/*K*_m_ of 1762 × 10^−5^ M s^−1^ L g^−1^ were determined for PCN-224(Fe), and these values are superior to a range of previously reported nanozymes with peroxidase-like activity (Table S1†).^69–71^ In addition, kinetics experiments were also carried out for the dual-functional AuNPs@PCN-224(Fe) with d-glucose. The Michaelis–Menten curves and Lineweaver–Burk analysis of the kinetic process are shown in Fig. 4c and d, respectively. The *V*_max_, *K*_m_, and *K*_w_/*K*_m_ values of AuNPs@PCN-224(Fe) were determined to be 0.06 μM s^−1^, 0.52 mM and 11.54 × 10^−5^ M s^−1^ L g^−1^, respectively. A comparison to previously known nanozymes with dual-catalytic activities indicates that our hybrid MOF is among the more active in terms of glucose sensing (Table S2†),^72–75^ because the majority of previously developed nanozymes require the presence of GOx to achieve the cascade reaction, whereas our AuNPs@PCN-224(Fe) is completely natural enzyme-free.

### Use of the nanozyme for d-glucose sensing

**Fig. 5:**
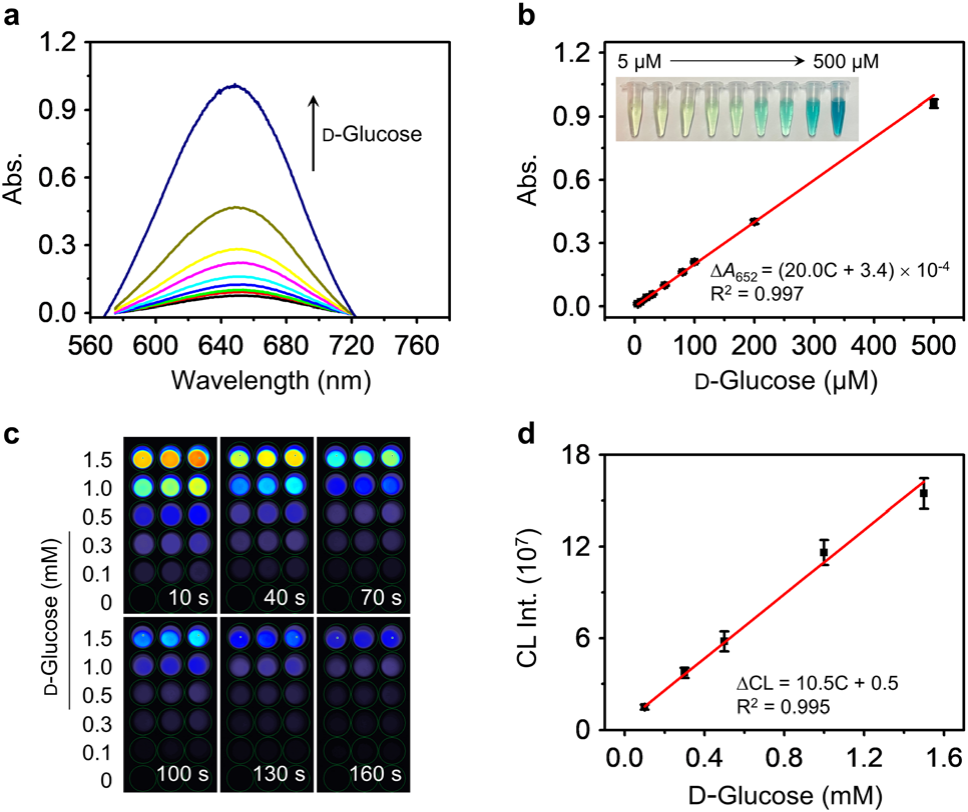
(a) UV-vis absorption spectra of TMB (5.0 mM in PBS solution) with increasing d-glucose concentrations (5–500 μM) catalyzed by AuNPs@PCN-224(Fe). (b) Plotting the absorption changes of TMB (at 652 nm) as a function of d-glucose concentrations (inset: photo of the TMB/AuNPs@PCN-224(Fe) solutions with different concentrations of d-glucose). (c) Chemiluminescence (CL) imaging of Luminol (1 mM in PBS solution) with increasing d-glucose concentrations catalyzed by AuNPs@PCN-224(Fe) (*n* = 3). (d) Plotting the CL intensity changes of Luminol as a function of d-glucose concentrations.

Finally, we evaluated the d-glucose sensing performances of the AuNPs@PCN-224(Fe) by both colorimetric and CL analyses, two commonly used methodologies clinically. Fig. 5a and b indicate that the concentration-dependent absorption enhancement of TMB with d-glucose as catalyzed by the dual-functional nanozyme. A good linearity was obtained over a concentration range from 5–500 μM, and a limit of detection (LOD) of 3.06 nM was determined. In compared to previously reported colorimetric systems, we found that our nanozyme was the most sensitive (Table S3†), noting that the sensitivity of our system surpasses those of other nanozymes that require the presence of GOx.^76–78^ In addition, a similar bifunctional nanozyme based on a Cu-TCPP nanosheet (use of Cu instead of Fe), in which AuNPs were *in situ* grown, was constructed previously.^46^ The linear range and LOD of the nanosheet for d-glucose were 10–300 μM and 8.5 μM, respectively. These values are below those of our AuNPs@PCN-224(Fe), suggesting the fine morphological control of MOFs as well as the use of Fe as the metallic center might contribute to enhancing glucose sensitive sensing.

Luminol, was then used for CL-based d-glucose sensing with AuNPs@PCN-224(Fe) as the catalyst. The test samples were placed in a black 96-well plate, and then the CL intensities were automatically read using a CL reader with the intensities collected at 10 s, 40 s, 70 s, 100 s, 130 s and 160 s. We found that the CL intensity of Luminol gradually enhanced with increasing concentrations of d-glucose, concomitantly with a corresponding transition in the image hue from blue to orange (Fig. 5c). The CL produced decreased with time, and was quenched after ∼160 s. This agrees with the typical emissive property of Luminol. The CL emission spectra of AuNPs@PCN-224(Fe) recorded on a Varian Cary Eclipse fluorescence spectrophotometer in the presence of different concentrations of d-glucose are shown in Fig. S18.† These preliminary results suggest the suitability of our nanozyme for developing a CL-based diagnostic assays. Plotting the CL intensity of Luminol as a function of d-glucose concentration produces a linear relationship from 0.1–1.5 mM (Fig. 5d).

Again, the sensitivity obtained from the CL assay was better than for previously reported nanozymes (Table S4†).^79–83^ Then, the selectivity of AuNPs@PCN-224(Fe) was evaluated. We determined that in the presence of 0.2 mM of other saccharides including d-fructose, maltose, saccharose, lactose, d-galactose, d-mannose, glucan and beta-cyclodextrin weaker signals than those with d-glucose were observed when using both the colorimetric (Fig. S19a†) and CL (Fig. S19b†) modes. The nanozyme was more selective in the CL assay than in the colorimetric test. The lack of selectivity by MOF-based nanozymes for glucose and galactose, a pair of C4–epimers, is a common issue that needs to be addressed.^37,73^ Natural enzymes possess structurally well-defined active pockets to distinguish monosaccharide epimerization; this exquisite feature suggests that supramolecular host systems^84^ should be incorporated that can accommodate only glucose (C4-equatorial bond) into MOF-based nanozymes.

Finally, the feasibility of the nanozyme for real-world applications was examined by measuring d-glucose concentrations in a serum-mimicking solution containing biological species commonly found in human blood.^55,85^ Different concentrations of d-glucose were added to the prepared solutions with a starting concentration of 50 μM, and then the final concentrations (total of the starting and added d-glucose concentrations) were quantified using AuNPs@PCN-224(Fe) with both TMB and Luminol as the substrate (Table S5†). Recovery rates ranging from 94.4–102.0% were determined for the colorimetric assay with relative standard deviations (RSD) of ≤5.5%. Likewise, for the CL assay, recovery rates ranging from 99.5–102.9% and an RSD of ≤5.1% were obtained. These results suggest the reliability of the nanozyme for d-glucose sensing.

## Conclusions

We have constructed an enzyme-free dual-functional nanozyme, AuNPs@PCN-224(Fe), through the *in situ* growth of AuNPs on a PCN-224(Fe)-based MOF for d-glucose sensing. The proximity of AuNPs and PCN-224(Fe) enables the sensitive cascade catalysis of d-glucose to generate H_2_O_2_*in situ* in the presence of oxygen, and then the oxidation of TMB or Luminol for colorimetric and CL-based analytical assays. The sensitivity of the system surpasses most previously reported nanozymes that rely on the incorporation of natural enzymes. The good stability, recyclability and reliability in quantitative assays suggest the practicability of our nanozyme for diagnostic applications. Our research also provides new insights into the construction of natural enzyme-free hybrid MOF systems for biosensing applications.

## Data availability

All experimental and data associated with this article have been included in the main text and ESI.†

## Author contributions

P.-H. T., and X.-P. H. conceived the project and designed the experiments. P.-H. T., and J.-J. W. performed the experimental work. P.-H. T., X.-L. H., and X.-P. H. analysed and interpreted the data. The manuscript was written by P.-H. T., and X.-P. H. and revised by P.-H. T., T. D. J., and X.-P. H. All of the authors contributed to the discussion of the results.

## Conflicts of interest

There are no conflicts to declare.

## Supplementary Material

SC-014-D3SC02598E-s001
