# Supplementary material for: Metal–organic framework (MOF) hybridized gold nanoparticles as a bifunctional nanozyme for glucose sensing
Source: Chem Sci. 2023 Jun 22;14(28):7762–9. doi: 10.1039/d3sc02598e (PMC10355114; doi:10.1039/d3sc02598e)
Supplement: SC-014-D3SC02598E-s001 [file SC-014-D3SC02598E-s001.pdf]

Electronic Supplementary Material (ESI) for Chemical Science.
This journal is © The Royal Society of Chemistry 2023

Supporting Information

Metal-organic framework (MOF) hybridized gold nanoparticles as a bi-functional nanozyme for glucose sensing

Pei-Hong Tong,[a] Jing-Jing Wang,[a] Xi-Le Hu,[a] Tony D. James*[c,d] and Xiao-Peng He*[a,b]

[a.] Key Laboratory for Advanced Materials and Joint International Research Laboratory of Precision Chemistry and Molecular Engineering, Feringa Nobel Prize Scientist Joint Research Center, School of Chemistry and Molecular Engineering, East China University of Science and Technology, 130 Meilong Rd., Shanghai 200237, China

[b.] The International Cooperation Laboratory on Signal Transduction, Eastern Hepatobiliary Surgery Hospital, National Center for Liver Cancer, Shanghai 200438, China

[c.] Department of Chemistry, University of Bath, Bath, BA2 7AY, UK

[d.] School of Chemistry and Chemical Engineering, Henan Normal University, Xinxiang 453007, China

*Corresponding authors.
xphe@ecust.edu.cn (X.-P.H.)
t.d.james@bath.ac.uk (T.D.J.)

CONTENTS LIST

S1. Experimental Section
S2. Additional Figures and Tables
S3. Additional References

S1. Experimental Section

Materials and Instrumentation. All purchased chemicals and reagents are of analytical grade. Tetra(4-carboxyphenyl)porphine (**TCPP**), $FeCl_2 \cdot 4H_2O$, $ZrClO_2 \cdot 8H_2O$, chloroauric acid, 3,3′,5,5′-tetramethylbenzidine (**TMB**, 99%), hydrogen peroxide (H_2O_2, 30%), horseradish peroxidase (HRP), glucose oxidase (GOx), D-fructose, D-galactose, D-mannose, glucan (Dextran, Mw 100,000), and D-glucose were purchased from Sigma-Aldrich. Maltose, saccharose, lactose and beta-cyclodextrin were purchased from Aladdin. Pro-light HRP Chemiluminescent Kit was purchased from TIANGEN. ^1H NMR spectra were recorded on a Bruker AM 400 MHz spectrometer with tetramethylsilane (TMS) as the internal reference. High-resolution mass spectra (HRMS) were recorded with a Waters Micromass LCT mass spectrometer. Transmission electron microscopy (TEM, Talos F200X) or field-emission scanning electron microcopy (SEM, Helios G4 UC) was used to characterize the morphology of materials, and energy-dispersive X-ray (EDX) spectroscopy was used for elemental mapping. The particle size and Zeta potential of materials were obtained from a Zeta sizer Nano ZS (Malvern Instruments). Fourier transform infrared (FT-IR) spectra were measured on a Thermo Scientific Nicolet 6700. Powder X-ray Diffraction Spectroscopy (PXRD) was performed using Rotating Anode X-ray Powder Diffractometer (Rigaku, Japan). N_2 sorption isotherms were collected using Tristar II 3020 surface area/pore size analyzer. The surface composition and chemical states of materials were analyzed by X-ray Photoelectron Spectroscopy (XPS) performed on the ESCALAB 250Xi X-ray photoelectron spectrometer (Thermo Scientific, USA) using monochromatic Al Kα radiation. Electron Spin Resonance (ESR) spectra were performed on the EMX-8/2.7 ESR spectrometer. UV-vis absorption spectra were measured on a Varian Cary 500 UV-Vis spectrophotometer or a UV microplate reader (DR-3000). Chemiluminescence was measured on a Fiji LAS-4000 Super CCD Remote Control Science Imaging System. Chemiluminescence spectra were obtained with a Varian Cary Eclipse fluorescence spectrophotometer.

Synthesis of Fe(II) meso-tetra(4-carboxyphenyl)porphine) (TCPP-Fe). A 100 mL two-necked, round-bottomed flask was charged with $FeCl_2 \cdot 4H_2O$ (530 mg, 2.7 mmol), **TCPP** (250 mg, 0.3 mmol), and DMF (35 mL). The mixture was heated at reflux for 12 h under an argon atmosphere in the dark. The reaction mixture was then cooled to room temperature and deionized H_2O (65 mL) was added to aid precipitation. The resulting precipitate was collected by suction filtration and washed with deionized H_2O. The solid was dissolved in an aqueous NaOH solution (0.1 M, 25 mL), and **TCPP-Fe** was precipitated by gradually adding HCl (1.0 M, 25 mL). Finally, the resulting precipitate was filtered, washed with deionized H_2O (5 × 25 mL), and dried in vacuum to give **TCPP-Fe** as a dark-purple powder (245 mg, 91.8% yield).

Synthesis of PCN-224(Fe), PCN-224, AuNPs and AuNPs@PCN-224(Fe). PCN-224(Fe) was synthesized according to a previous report.[1] In brief, $ZrOCl_2 \cdot 8H_2O$ (90 mg), **TCPP-Fe** (30 mg), and benzoic acid (870 mg) were dissolved in DMF (30 mL). Then, the mixture was heated at 90 °C under magnetic stirring (300 rpm) for 5 h. The reaction mixture was cooled to room temperature, centrifuged (12000 rpm, 15 min), and washing with DMF five times. **PCN-224** was synthesized similarly with **TCPP** (27 mg) used instead of **TCPP-Fe**.

AuNPs were synthesized according to a previous report.[2] In brief, $HAuCl_4$ trihydrate (0.05 mmol) and PVP (20 mg) were mixed in 45 mL deionized H_2O. After incubation for 10 min, a freshly-prepared $NaBH_4$ solution (100 mM, 5 mL) was rapidly injected. The reaction mixture was then stirred at room temperature for 24 h to obtain AuNPs with an average size of 10 nm.

To construct the hybrid nanozyme, **PCN-224(Fe)** (10 mg) was dispersed in 10 mL of deionized H_2O under sonication. Then, an aqueous solution of $HAuCl_4$ (10 mM, 1 mL) was added dropwise into the vial under vigorous stirring. The vial was sealed and stirred vigorously for 12 h in the dark. Then, the resulting precipitates were collected by centrifugation (12000 rpm, 15 min) and washed twice with deionized H_2O. The precipitates were redispersed into 10 mL of deionized H_2O. Next, a freshly-prepared, ice-cold aqueous solution of $NaBH_4$ (0.1 M, 250 μL) was quickly injected followed by a vigorous stirring for another 30 min. Finally, **AuNPs@PCN-224(Fe)** were obtained as a fuchsia solid after centrifugation (12000 rpm, 15 min) and then washing with deionized H_2O five times.

Electron spin resonance (ESR) experiment. 5,5-dimethyl-1-pyrroline-*N*-oxide (DMPO) was used as the spin trapping agent. In a typical measurement, 10 μL of **AuNPs@PCN-224(Fe)** aqueous suspension (final concentration 100 mg L^{-1}) and 10 μL of 500 mM D-glucose were diluted with 380 μL of phosphate-buffered saline (PBS, 0.1 M, pH 7.4). Immediately, 100 μL of 100 mM DMPO was added into the above mixture. The resultant solution was extracted by quartz capillary tube and placed in a glass tube. After 5 min, the spectrum was recorded on a ESCALAB 250Xi.

Measuring the peroxidase-like activity of PCN-224(Fe). To a PBS (0.1 M, pH 7.4) of **PCN-224(Fe)** (100 μg mL^{-1}), **TMB** (5 mM, used as substrate) and H_2O_2 (5 mM) were added. After the mixture was incubated for 10 min, the absorption of **TMB** was measured by a Varian Cary 500 UV-Vis spectrophotometer or a UV microplate reader (DR-3000). The peroxidase activity of **PCN-224** and HRP was measured by the same protocol.

Measuring the cascade catalytic activities of AuNPs@PCN-224(Fe). To a PBS (0.1 M, pH 7.4) of **AuNPs/PCN-224(Fe)** (1 mg mL^{-1}), D-glucose (0.2 M) and **TMB** (5.0 mM) were added. After the mixture was incubated for 40 min, The absorption of **TMB** was measured by a Varian Cary 500 UV-Vis spectrophotometer or a UV microplate reader (DR-3000). For the selectivity assay, D-fructose, maltose, saccharose, lactose, D-galactose, D-mannose, glucan (Dextran, Mw 100,000) and Beta-cyclodextrin (0.2 M) were used separately instead of D-glucose. The cascade catalytic activities of AuNPs, **PCN-224(Fe)**, GOx, HRP, mixture of COx and HRP, and mixture of AuNPs and **PCN-224(Fe)** were measured by the same protocol. For the chemiluminescent assay, **Luminol** (1 mM) was used as the substrate instead of **TMB**, and the chemiluminescent signals were measured on a Fiji LAS-4000 Super CCD Remote Control Science Imaging System.

To test the recyclability of the bifunctional nanozyme, **AuNPs/PCN-224(Fe)** was recovered by centrifugation (12 000 rpm, 2 min) after one catalytic cycle (40 min) and its concentration was calibrated by UV-vis spectroscopy. This action was repeated five times.

A serum-like solution was prepared according to the literature[3,4]. A mixture of 0.14 M sodium chloride, 4.2 mM sodium bicarbonate, 3.0 mM potassium chloride, 1.5 mM magnesium chloride, 2.6 mM calcium chloride, 1.2 mM dibasic potassium phosphate, 0.5 mM sodium sulfate, 5 mM D-glucose, 5 mM cholesterol, 20 μM bilirubin, 3.0 mM of common blood plasma amino acids (glutamine, glycine, valine, arginine, lysine and alanine at equimolar concentrations of 0.5 mM) and 35 g L^{-1} human serum albumin was dissolved in deionized water. the pH of the resulting solution was adjusted to 7.40 with HCl (1.0 M). Then, test solutions were diluted in distilled water to ensure that the D-glucose concentrations were within the linear range of the calibration curve and to reduce possible matrix effects. Recoveries were also determined in the same samples by spiking the raw (undiluted) samples with different concentrations of D-glucose. Then, the final concentrations of D-glucose in the spiked samples were detected by the above colorimetric or chemiluminescent detection procedure. All tests were repeated five times.

Kinetics study. The reaction kinetics of the nanozymes was measured using **TMB** as the substrate. For the peroxidase-like catalytic reaction, **PCN-224(Fe)** (1 mg mL^{-1}) was added into a PBS (0.1 M, pH 7.4) containing 10 mM H_2O_2 and **TMB** of different concentrations (0.0125, 0.025, 0.05, 0.1, 0.3, 0.5 and 0.8 mM). Then, the absorbance at 652 nm of the solutions was measured in time-course mode on a UV microplate reader (DR-3000). For the cascade reaction, **AuNPs@PCN-224(Fe)** (1 mg mL^{-1}) was added into a PBS (0.1 M, pH 7.4) containing 5 mM **TMB** and D-glucose of different concentrations (0.05, 0.1, 0.3, 0.5, 1, 2 and 3 mM). Then, the absorbance at 655 nm of the solutions was measured in time-course mode on a UV microplate reader (DR-3000).

S2. Additional Figures and Tables

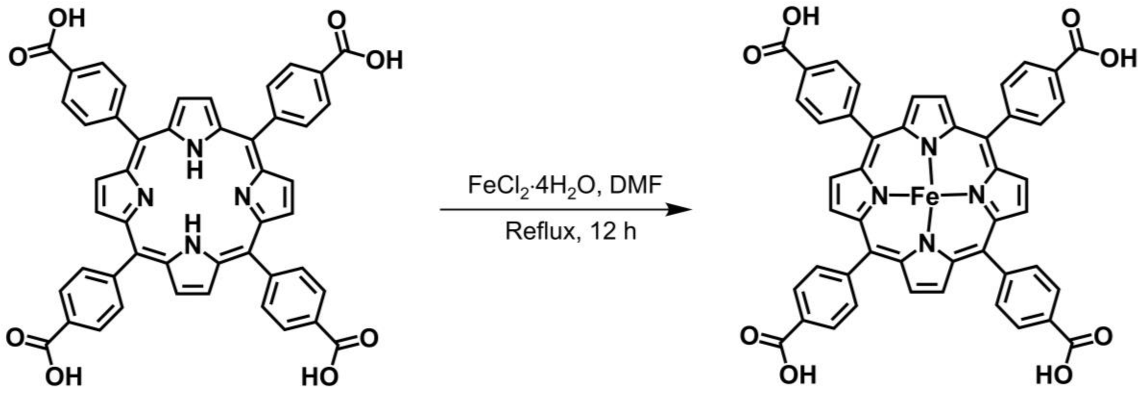

Scheme S1. Synthetic procedure of **TCPP-Fe**.

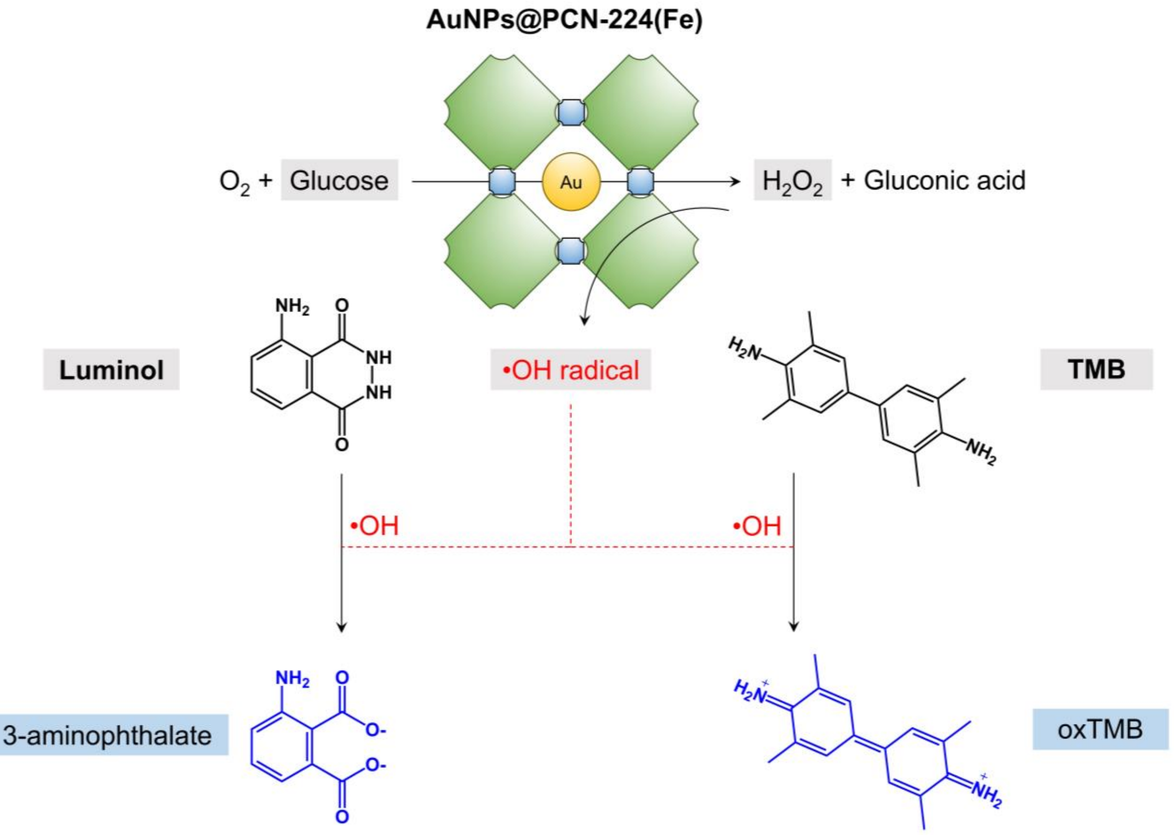

Scheme S2. Plausible mechanism by which **AuNPs@PCN-224(Fe)** catalyzes the colorimetric and chemiluminescent reaction of **TMB** and **Luminol**, respectively.

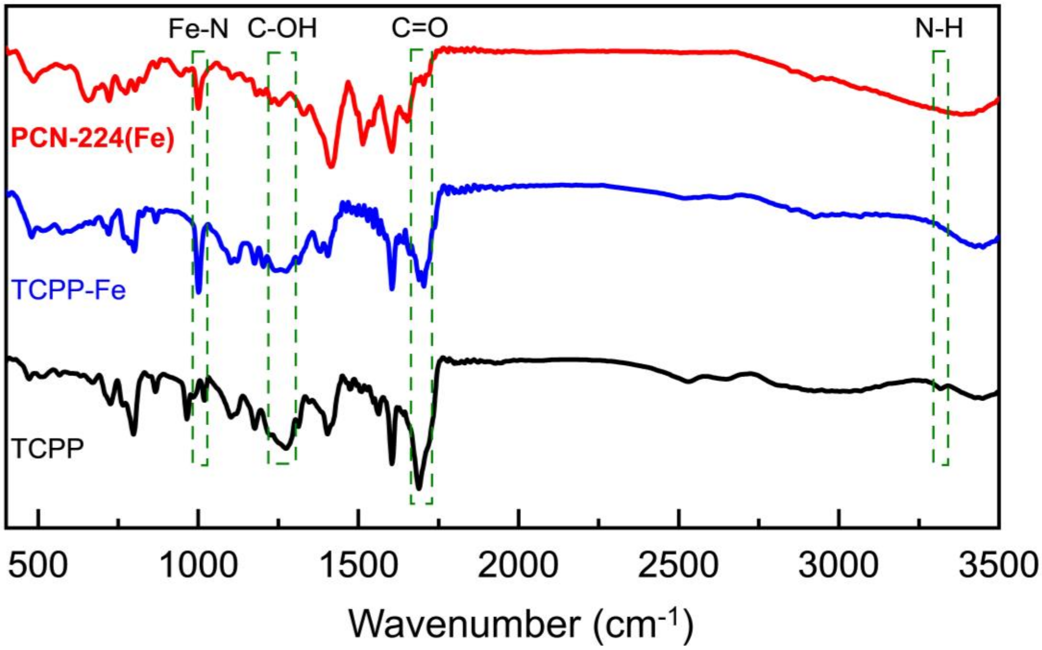

Fig. S1. Stacked FTIR spectra of **TCPP**, **TCPP-Fe** and **PCN-224(Fe)**.

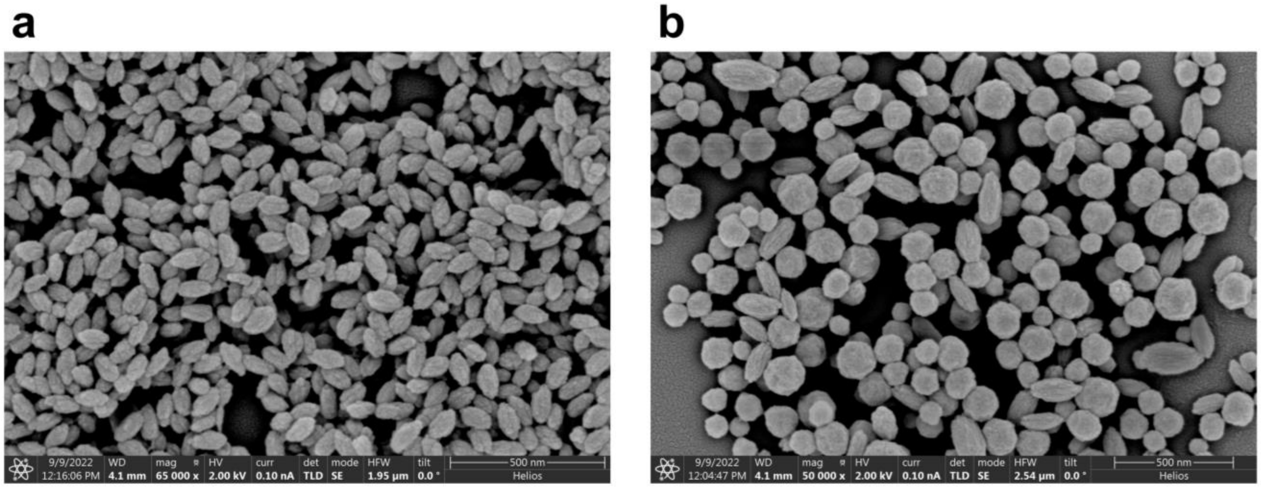

Fig. S2. SEM images of (a) **PCN-224(Fe)** and (b) **PCN-224**.

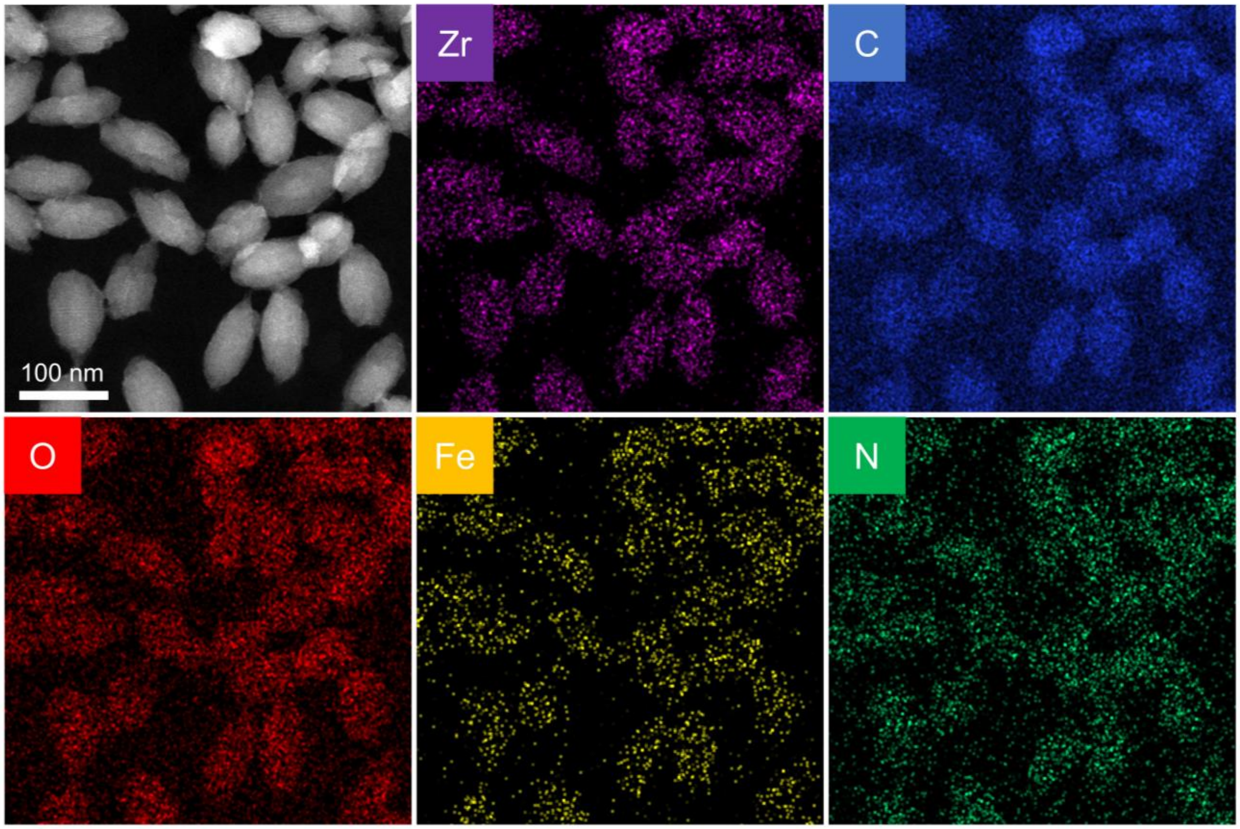

Fig. S3. Dark-field TEM image of **PCN-224(Fe)** and its corresponding EDC mapping images. The Zr element belong to the Zr_6 cluster, the C, O and N elements belong to **TCPP**, and the Fe element belongs to Fe^{2+} coordinated in the porphyrin ring.

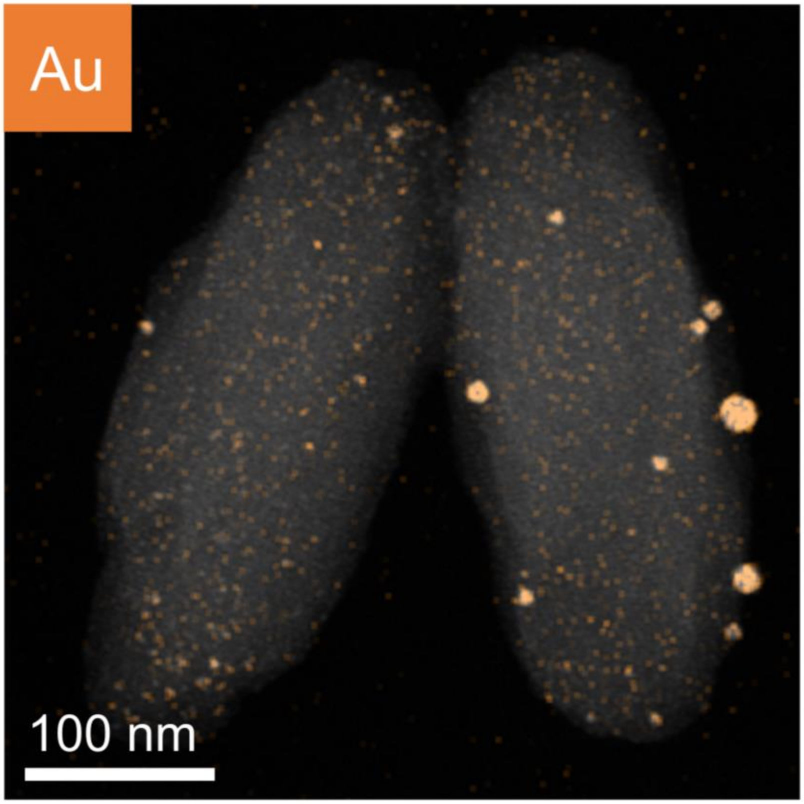

Fig. S4. Dark-field TEM image of **AuNPs@PCN-224(Fe)**. The orange dots represent AuNPs, which are uniformly distributed in **PCN-224(Fe)** in general; however, particles with larger sizes could be seen on the surface of the MOFs.

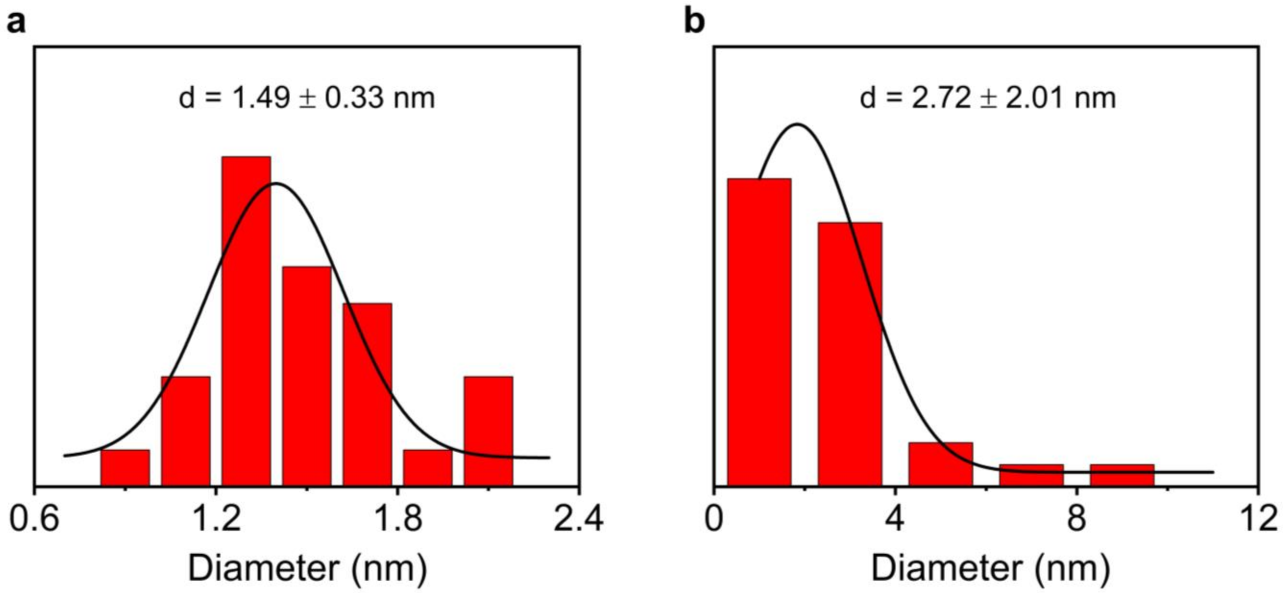

Fig. S5. (a) Particle size distribution of AuNPs embedded in **PCN-224(Fe)** determined from Fig. 2c. (b) Particle size distribution of AuNPs embedded in and adsorbed on the surface of **PCN-224(Fe)** determined from Fig. S4.

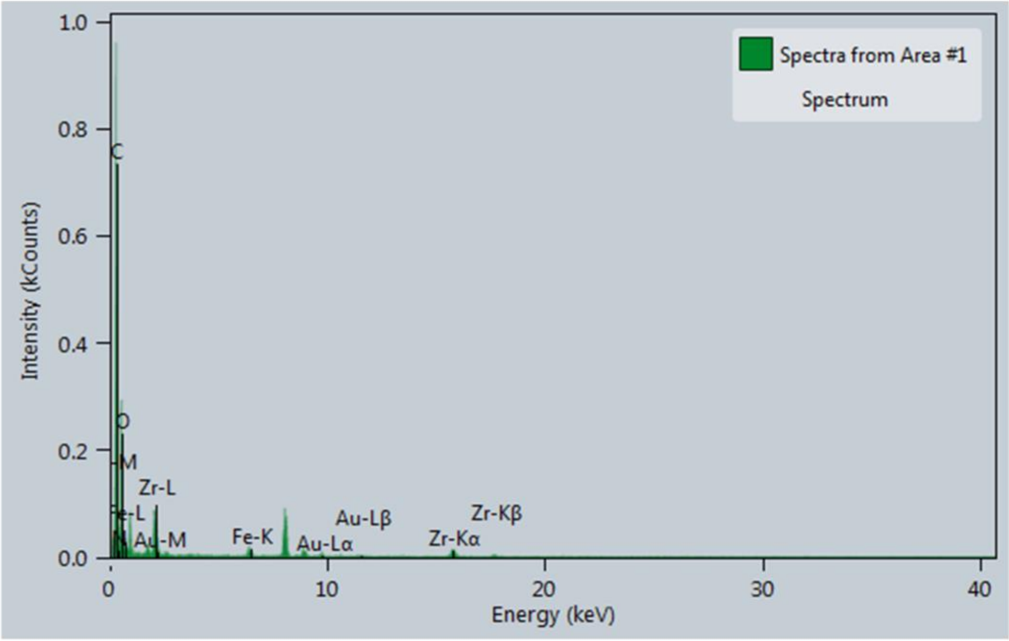

Element	Family	Mass Fraction (%)	Mass Error (%)	Fit error (%)
C	K	66.84	2.50	1.03
N	K	0.89	0.24	17.42
O	K	15.38	3.13	0.51
Fe	K	2.06	0.29	2.40
Zr	K	12.87	1.72	1.97
Au	L	1.96	0.25	5.07

Fig. S6. Energy-dispersive X-ray (EDX) spectrum and quantitative analysis of elemental distribution of **AuNPs@PCN-224(Fe)**.

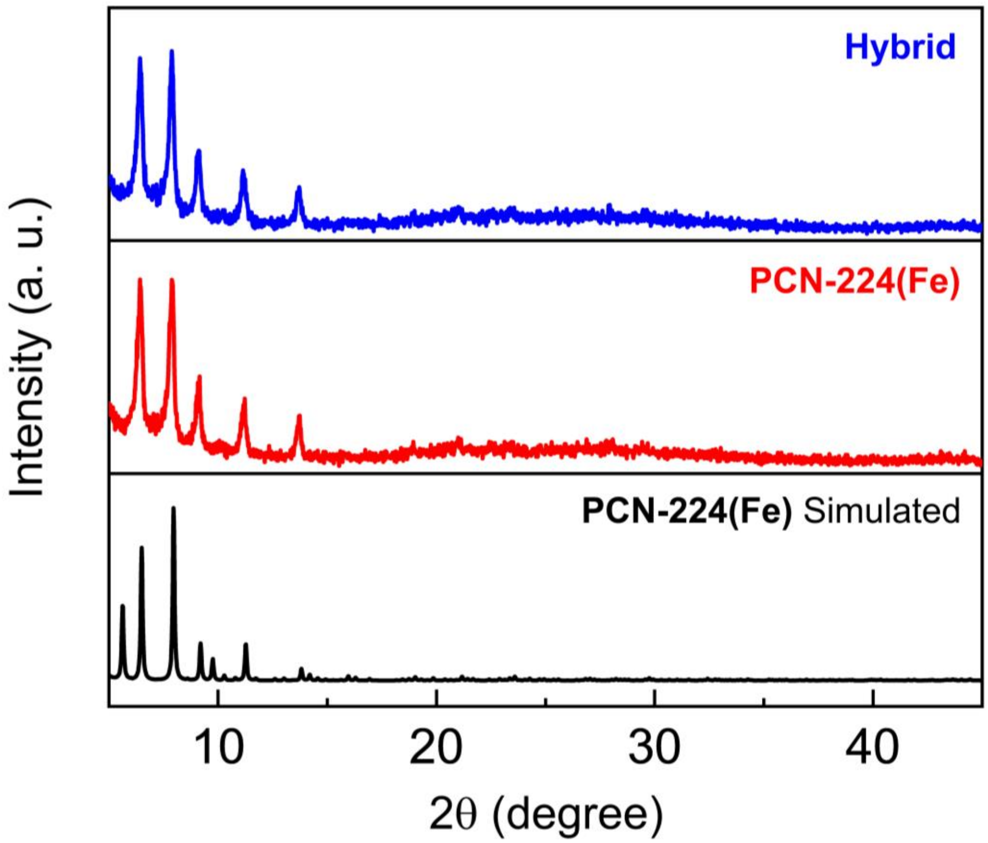

Fig. S7. PXRD patterns of simulated **PCN-224(Fe)**, synthetic **PCN-224(Fe)**, and **Hybrid**.

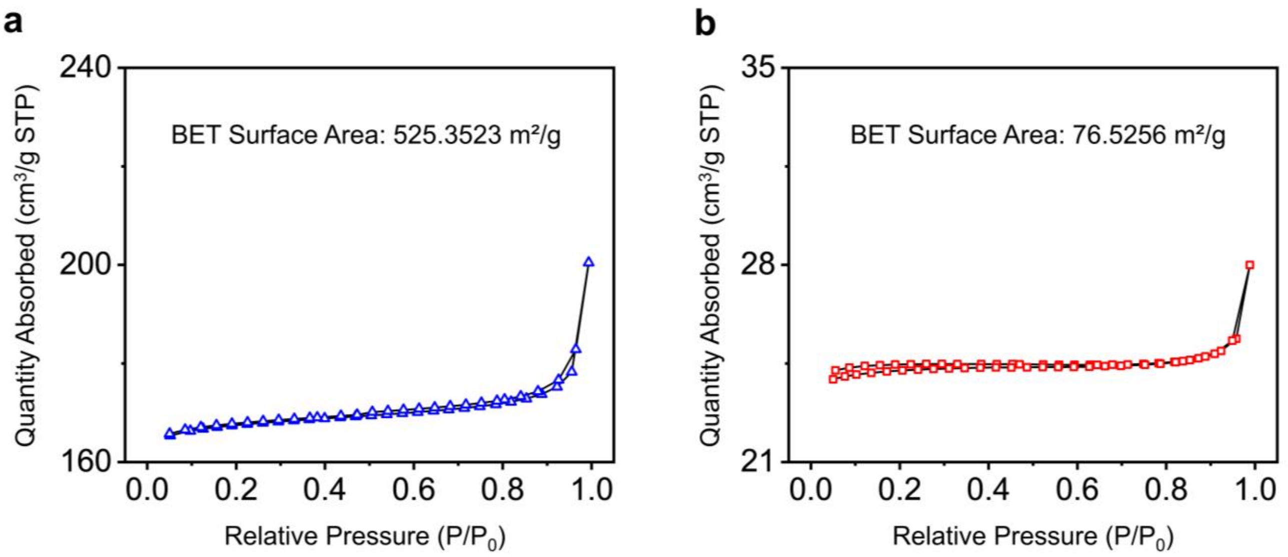

Fig. S8. Nitrogen adsorption isotherms of (a) **PCN-224(Fe)** and (b) **Hybrid** at 77K.

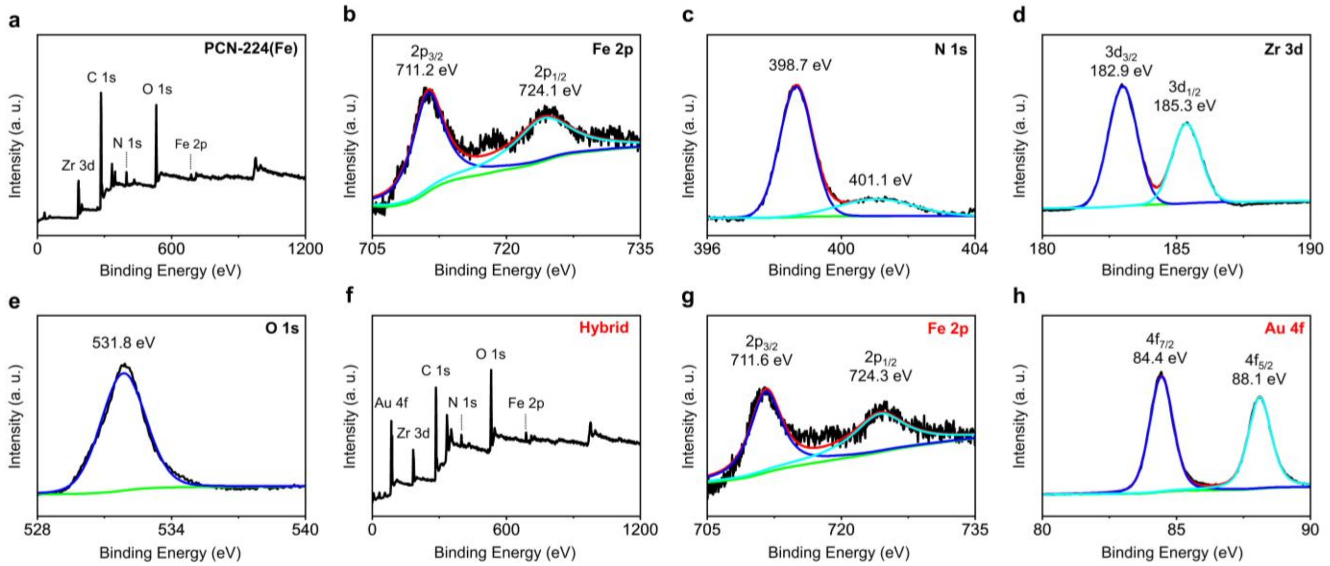

Fig. S9. Survey XPS spectrum of (a) **PCN-224(Fe)** and the corresponding high-resolution XPS spectrum of (b) Fe 2p, (c) N 1s, (d) Zr 3d, and (e) O 1s. Survey XPS spectrum of (f) **Hybrid** and the corresponding high-resolution XPS spectrum of (g) Fe 2p, (h) Au 4f.

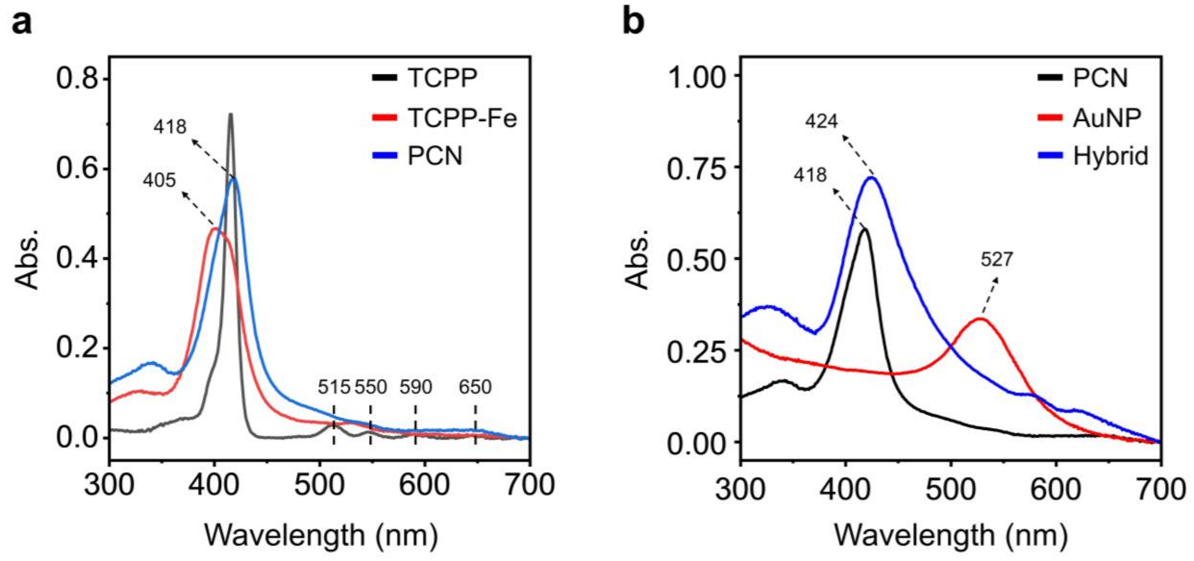

Fig. S10. (a) Stacked UV–vis absorption spectra of **TCPP** (10 μM), **TCPP-Fe (**5 μg mL^{-1}**)** and **PCN-224(Fe)** (10 μg mL^{-1}) in MeOH. (b) UV-vis absorption spectra of AuNPs (20 μg mL^{-1}), **PCN-224(Fe)** (10 μg mL^{-1}) and the **Hybrid** nanozyme (10 μg mL^{-1}).

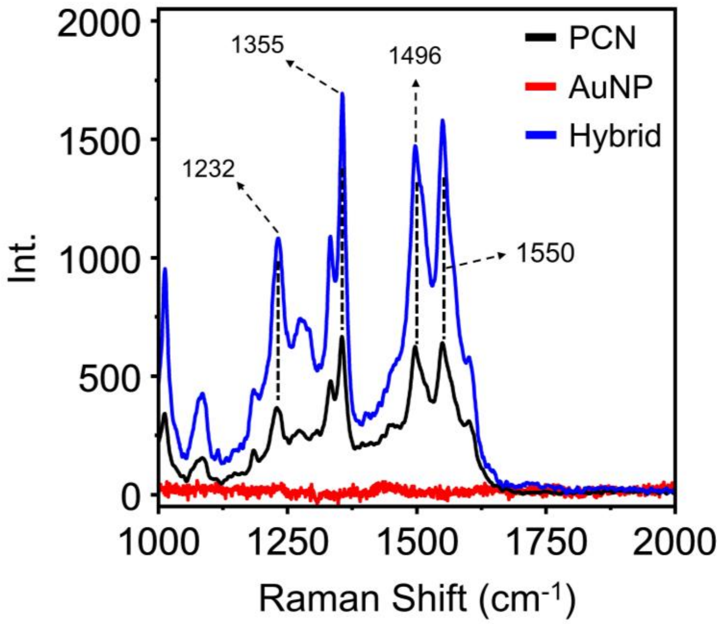

Fig. S11. SERS spectra of AuNPs, **PCN-224(Fe)** and the **Hybrid** nanozyme.

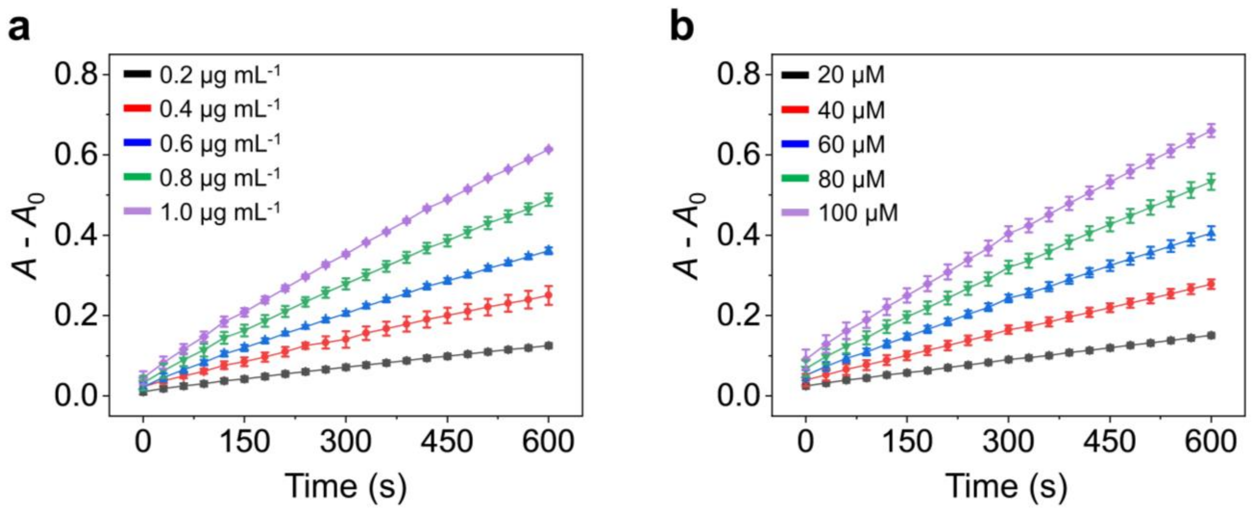

Fig. S12. (a) Plotting the absorption changes of **TMB** (1 mM) with different indicated concentrations of **PCN-224(Fe)** in the presence of H_2O_2 (0.1 mM) as a function of time. (b) Plotting the absorption changes of **TMB** (1 mM) with different indicated concentrations of H_2O_2 in the presence of **PCN-224(Fe)** (1 μg mL^{-1}) as a function of time. A and A_0 are the absorption of **TMB** in the absence and presence of analytes, respectively. Error bars mean standard deviation (n = 3).

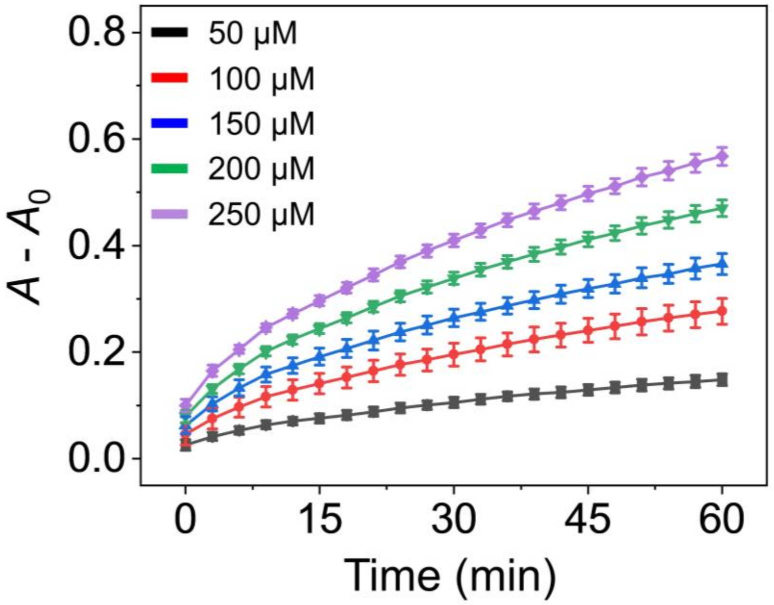

Fig. S13. Plotting the absorption changes of **TMB** (5 mM) with different indicated concentrations of D-glucose in the presence of **Hybrid** (1 mg mL^{-1}) as a function of time. A and A_0 are the absorption of **TMB** in the absence and presence of analytes, respectively. Error bars mean standard deviation (n = 3).

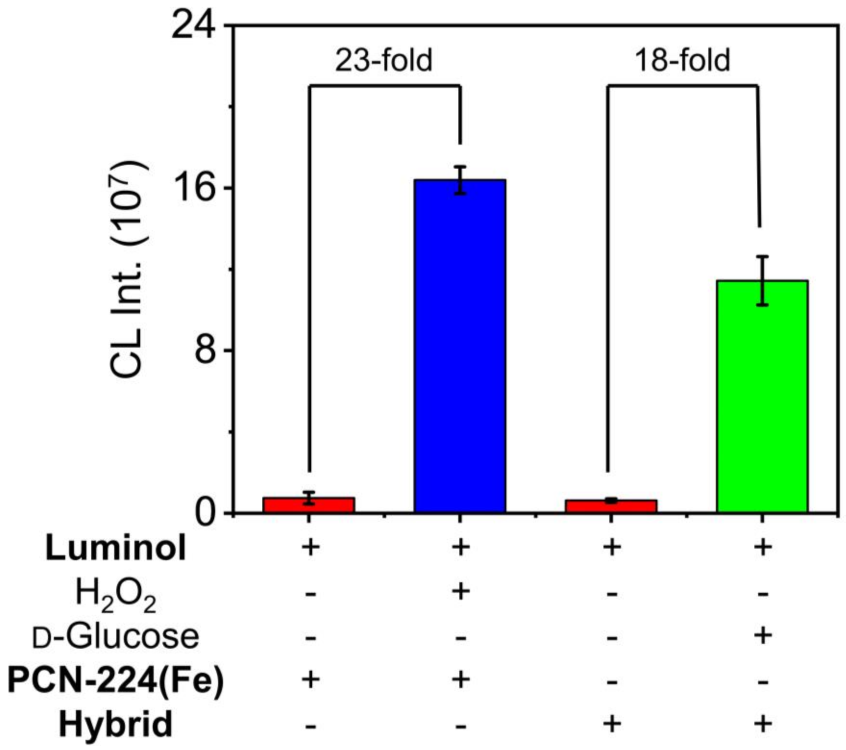

Fig. S14. Measuring the peroxidase-like activity of **PCN-224(Fe)** and cascade catalytic activities of **Hybrid** by measuring the CL intensity of **Luminol** in PBS solution under different indicated conditions. The concentrations used for H_2O_2, **Luminol**, D-glucose, **PCN-224(Fe)** and **Hybrid** are 5 mM, 5 mM, 1 mM, 1 mg mL^{-1}, and 1 mg mL^{-1}, respectively.

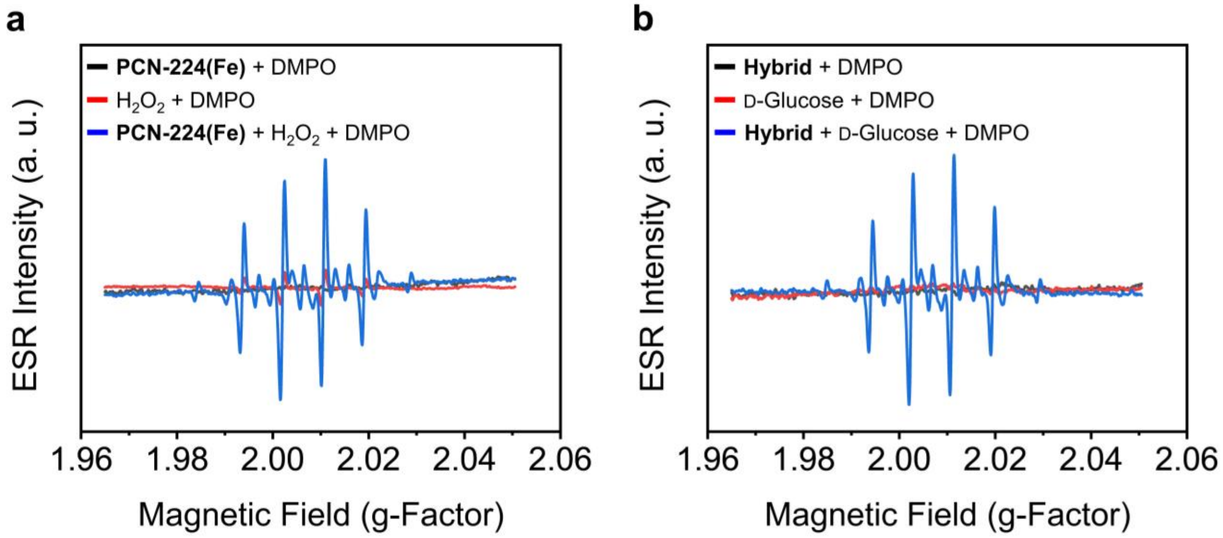

Fig. S15. (a) ESR spectra of •OH radicals determined from the **PCN-224(Fe)**/DMPO, H_2O_2/DMPO and **PCN-224(Fe)**/H_2O_2/DMPO systems. (b) ESR spectra of •OH radicals determined from the **Hybrid**/DMPO, D-glucose/DMPO and **Hybrid**/D-glucose/DMPO systems. Conditions: 20 mM DMPO, 100 mg L^{-1} PCN-224(Fe), 100 mg L^{-1} **Hybrid**, 10 mM H_2O_2, and 10 mM D-glucose.

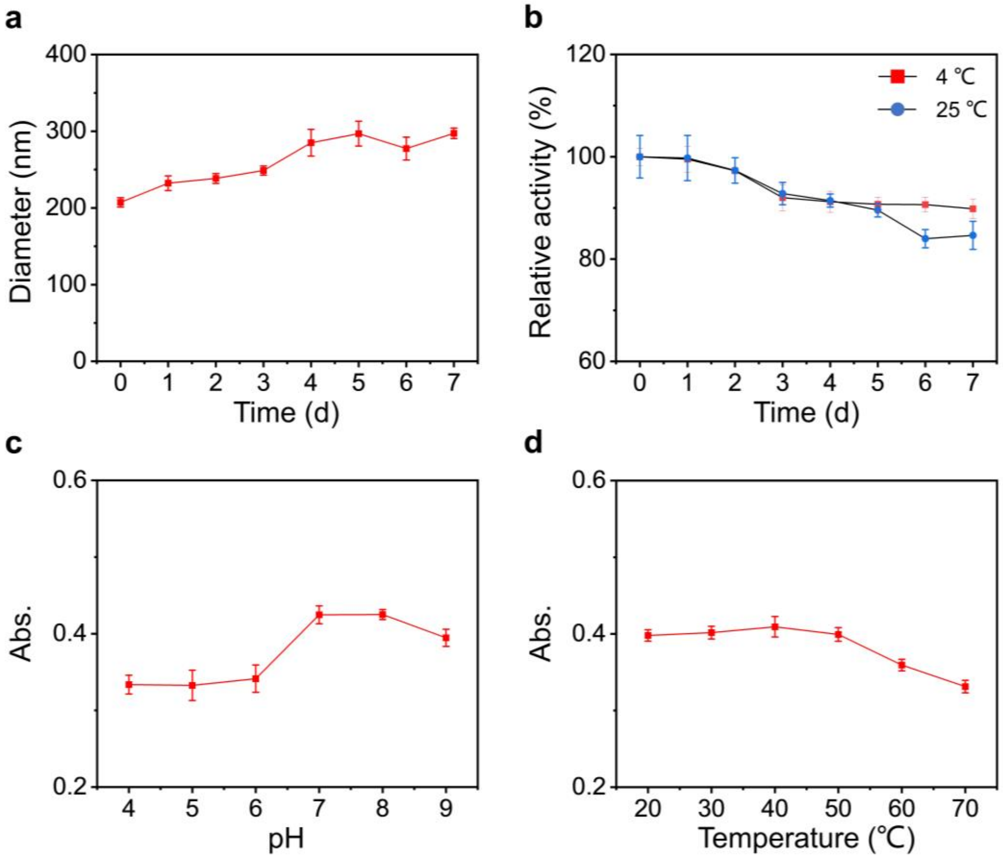

Fig. S16. (a) Particle size changes of **Hybrid** stored in PBS (pH 7.4) over time. (b) Catalytic activity changes of **Hybrid** stored in PBS (pH 7.4) at 4 °C and 25 °C. Measurement of the catalytic activity of **Hybrid** as a function of (c) pH, and (d) Temperature. Analysis of the catalytic activity of **Hybrid** by measurement of the UV-vis absorbance (at 652 nm) of **TMB** in PBS solution under the various indicated conditions. The concentrations used for **Hybrid**, **TMB** and D-glucose were 1 mg mL^{-1}, 5 mM and 0.2 mM, respectively.

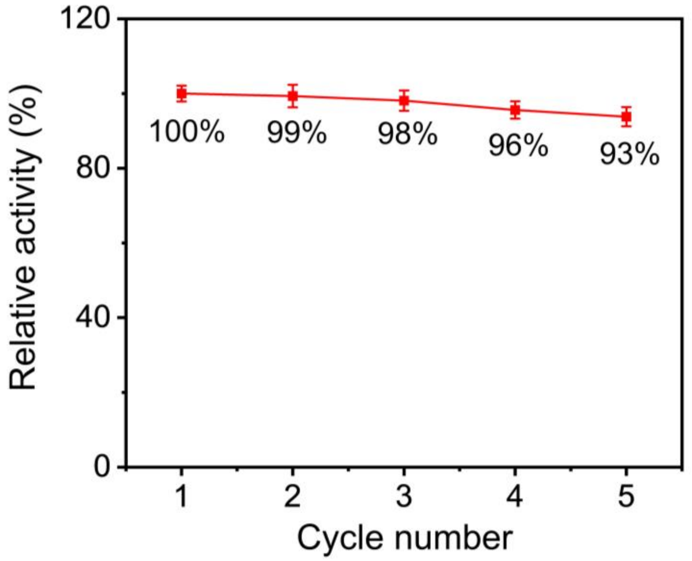

Fig. S17. Measuring the catalytic activity of **AuNPs@PCN-224(Fe)** after being recycled for one to five times. Note that the concentration of the nanozyme was calibrated before each test to avoid bias from simple quantity loss of the material during recovery.

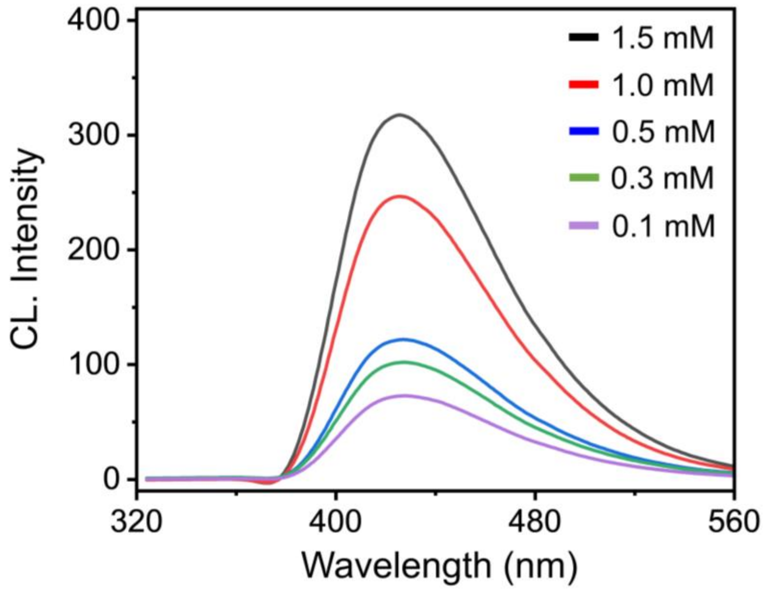

Fig. S18. CL spectra for **Luminol** (1 mM) with **AuNPs@PCN-224(Fe)** (1 mg mL^{-1}) in PBS solution in the presence of increasing concentrations of D-glucose.

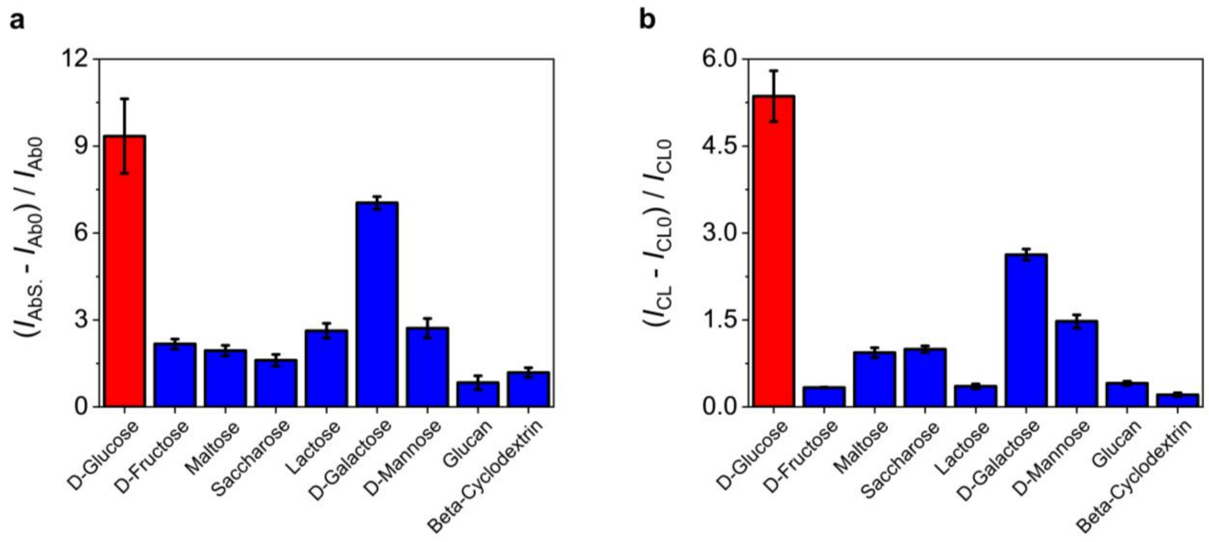

Fig. S19. (a) Enhancement of the absorption of **TMB** (5 mM) with **AuNPs@PCN-224(Fe)** (1 mg mL^{-1}) in the presence of different saccharides (0.2 mM). (b) Enhancement of the CL intensity of **Luminol** (1 mM) with **AuNPs@PCN-224(Fe)** (1 mg mL^{-1}) in the presence of different saccharides (0.1 mM). Error bars mean standard deviation (n = 3).

Table S1. Comparison of kinetic parameters of different nanozymes.

Catalyst	Substrate	K_m (mM)	V_{max} ($\times10^{-8}$ M s^{-1})	K_w ($\times10^{-8}$ M s^{-1} L g^{-1})	K_w/K_m	Ref.
Fe_3O_4 MNP	**TMB**	0.098	3.44	86	877.55	5
Fe-MIL-88NH_2		0.284	10.47	0.26	0.92	6
MIL-100(Fe)		0.424	2.10	10.5	24.76	7
2D Fe-BTC		0.261	7.95	0.79	3.03	8
PtNPs/Cu-TCPP(Fe)		0.044	16.51	75	1704.55	9
**PCN-224(Fe)**		0.039	68.70	68.7	1761.54	This work

Table S2. Comparison of kinetic parameters of different nanozymes.

Catalyst	Substrate	K_m (mM)	V_{max} ($\times10^{-8}$ M s^{-1})	K_w ($\times10^{-8}$ M s^{-1} L g^{-1})	K_w/K_m	Ref.
GOx@HP-MIL-88B-BA	D-Glucose / **TMB**	0.37	20.00	66.7	180.3	10
GOx&AuNCs@ZIF-8		0.40	2.00	0.15	0.38	11
GOx@Fe-BTC		3.30	2.02	0.08	0.03	12
Citric acid-based AuNPs		6.97	63.00	37.5	53.8	13
**AuNPs@PCN-224(Fe)**		0.52	6.00	6	11.54	This work

Table S3. Comparison of the sensitivity of different colorimetric methods for D-glucose sensing.

Catalyst	Linear range (μM)	Limit of detection (μM)	Ref.
GOx/hemin@ZIF-8	0-240	1.7	14
GOx@Fe-BTC	5-100	2.4	12
GOx@HP-MIL-88B-BA	2-100	0.98	10
Au@Pt core-shell nanorods	45-400	45	15
GOx/Co_3O_4@CeO_2	1-75	1.9	16
**AuNPs@PCN-224(Fe)**	5-500	0.003	This work

Table S4. Comparison of the sensitivity of different chemiluminescent methods for D-glucose sensing.

Catalyst	Linear range (μM)	Limit of detection (μM)	Ref.
GOx/CuO NPs	5-60	2.9	17
GOx/Cu-MOF	0.6-20	Not available	18
Gold colloid@HRP	10-1000	5	19
GOx/Cu-Doped Carbon Dots	1-48	0.32	20
GOx/MIL-53(Fe)	0.1-100	0.05	21
**AuNPs@PCN-224(Fe)**	100-1500	0.147	This work

Table S5. Recovery rate of D-glucose added to a serum-like solution determined by the colorimetric and chemiluminescent assays using **AuNPs@PCN-224(Fe)** as the catalyst.

Substrate	Samples (μM)	Added (μM)	Found (μM)	Recovery rate (%)	RSD[a] (%, n = 5)
**TMB** (Colorimetric)	50.0	50.0	97.2	94.4	5.5
		100.0	151.2	101.2	2.7
		200.0	254.0	102.0	1.6
**Luminol** (Chemiluminescent)	50.0	100.0	152.9	102.9	5.1
		200.0	248.9	99.5	2.3
		500.0	561.6	102.3	2.9

[a] RSD = Relative standard deviation.

S3. ADDITIONAL REFERENCES

1 J. Hu, W. Wu, Y. Qin, C. Liu, P. Wei, J. Hu, P. H. Seeberger and J. Yin, *Adv. Funct. Mater.* **2020**, *30*, 1910084.

2 J. Chen, Q. Ma, M. Li, D. Chao, L. Huang, W. Wu, Y. Fang and S. Dong, *Nat. Commun.* **2021**, *12*, 3375.

3 T. G. Choleva, V. A. Gatselou, G. Z. Tsogas and D. L. Giokas, *Microchim. Acta* **2018**, *185*, 1-9.

4 R. A. de Oliveira, F. Camargo, N. C. Pesquero and R. C. Faria, *Anal. Chim. Acta* **2017**, *957*, 40-46.

5 L. Gao, J. Zhuang, L. Nie, J. Zhang, Y. Zhang, N. Gu, T. Wang, J. Feng, D. Yang and S. Perrett, *Nat. Nanotechnol.* **2007**, *2*, 577-583.

6 Y. L. Liu, X. J. Zhao, X. X. Yang and Y. F. Li, *Analyst* **2013**, *138*, 4526-4531.

7 A. H. Valekar, B. S. Batule, M. I. Kim, K.-H. Cho, D.-Y. Hong, U.-H. Lee, J.-S. Chang, H. G. Park and Y. K. Hwang, *Biosens. Bioelectron.* **2018**, *100*, 161-168.

8 A. Yuan, Y. Lu, X. Zhang, Q. Chen and Y. Huang, *J Mater. Chem. B,* **2020**, *8*, 9295-9303.

9 H. Chen, Q. Qiu, S. Sharif, S. Ying, Y. Wang and Y. Ying, *ACS Appl. Mater. Interfaces* **2018**, *10*, 24108-24115.

10 Z. Zhao, Y. Huang, W. Liu, F. Ye and S. Zhao, *ACS Sustain. Chem. Eng.* **2020**, *8*, 4481-4488.

11 J. Chi, M. Guo, C. Zhang, Y. Zhang, S. Ai, J. Hou, P. Wu and X. Li, *New J. Chem.* **2020**, *44*, 13344-13349.

12 Z. Zhao, J. Pang, W. Liu, T. Lin, F. Ye and S. Zhao, *Microchim. Acta* **2019**, *186*, 1-8.

13 A. R. Deshmukh, H. Aloui and B. S. Kim, *Chem. Eng. J.* **2021**, *421*, 127859..

14 H. Cheng, L. Zhang, J. He, W. Guo, Z. Zhou, X. Zhang, S. Nie and H. Wei, *Anal. Chem.* **2016**, *88*, 5489-5497.

15 J. Liu, X. Hu, S. Hou, T. Wen, W. Liu, X. Zhu, J.-J. Yin and X. Wu, *Sens. Actuators B Chem.* **2012**, *166*, 708-714.

16 D. Jampaiah, T. S. Reddy, V. E. Coyle, A. Nafady and S. K. Bhargava, *J. Mater. Chem. B* **2017**, *5*, 720-730.

17 W. Chen, L. Hong, A.-L. Liu, J.-Q. Liu, X.-H. Lin and X.-H. Xia, *Talanta* **2012**, *99*, 643-648.

18 H. Yang, J. Liu, X. Feng, F. Nie and G. Yang, *Anal. Bioanal. Chem.* **2021**, *413*, 4407-4416.

19 D. Lan, B. Li and Z. Zhang, *Biosens. Bioelectron.* **2008**, *24*, 934-938.

20 Y. Duan, Y. Huang, S. Chen, W. Zuo and B. Shi, *ACS Omega* **2019**, *4*, 9911-9917.

21 X. Yi, W. Dong, X. Zhang, J. Xie and Y. Huang, *Anal. Bioanal. Chem.* **2016**, *408*, 8805-8812.
